# Tumour-associated macrophage infiltration differs in meningioma genotypes, and is important in tumour dynamics

**DOI:** 10.1186/s13046-025-03419-2

**Published:** 2025-05-27

**Authors:** Ting Zhang, Claire L. Adams, Gyorgy Fejer, Emanuela Ercolano, Jonathan Cutajar, Juri Na, Felix Sahm, C. Oliver Hanemann

**Affiliations:** 1 Peninsula Medical School, Faculty of Health, University of Plymouth, Plymouth, Devon, PL6 8BU UK; 2 Institute of Cancer, Affiliated Hospital of Jiangnan University, Wuxi, Jiangsu 214062 China; 3 School of Biomedical Sciences, Faculty of Health, University of Plymouth, Plymouth, Devon, PL6 8BU UK; 4 Derriford Hospital, University Hospitals Plymouth NHS Trust, Plymouth, Devon, PL6 8DH UK; 5 Department of Neuropathology, Institute of Pathology, University Hospital Heidelberg, 69120 Heidelberg, Germany

**Keywords:** Meningioma, Tumour microenvironment, Genotype, Methylation class, Macrophage-to-microglia ratio, Tumour-associated macrophages, 3D co-culture model, Tumour progression

## Abstract

**Background:**

Meningiomas are the most common primary intracranial tumours, with clinical behaviours ranging from benign to highly aggressive forms. The World Health Organisation classifies meningiomas into various grades, guiding prognosis and treatment. While surgery is effective for low-grade meningiomas, certain grade 1 tumours, as well as grade 2, 3, and recurrent cases are more aggressive and require new therapeutic approaches. Immunotherapy shows promise, with early-stage clinical trials demonstrating encouraging results. The tumour microenvironment (TME), particularly tumour-associated macrophages (TAMs), plays a pivotal role in tumour progression. TAMs influence tumour growth, metastasis, and immune evasion. However, their role in meningiomas, especially in relation to genomic mutations, remains poorly understood. Understanding how genetic alterations affect the TME is critical for developing targeted immunotherapies.

**Methods:**

This study employed multiplex immunohistochemistry and bulk RNA sequencing to explore immune infiltration in genetically stratified meningioma tissues and matched three-dimensional (3D) spheroid models. We compared immune cell populations across parental tissues, two-dimensional (2D) monolayer cultures, and 3D spheroid models. In addition, co-culture experiments were conducted, introducing M2-polarised macrophages derived from peripheral blood mononuclear cells to study the interactions between immune cells and tumour cells.

**Results:**

Our findings revealed significant differences in the immune infiltration patterns associated with specific genotypes and methylation classes, especially M2-like TAMs. Notably, the 3D spheroid models more closely replicated the TME observed in parental tissues compared to traditional 2D monolayer cultures, offering a superior platform for immune infiltration studies. Furthermore, co-culture experiments demonstrated that M2-polarised macrophages could effectively infiltrate tumour cells, promote tumour cell proliferation while inhibiting invasion, suggesting IL-6-mediated signalling in tumour progression.

**Conclusions:**

These findings suggest that 3D co-culture models offer an excellent system for studying the role of immune cells, specifically TAMs, in meningioma progression. By providing a more accurate representation of the TME, these models can help identify novel immunotherapy strategies aimed at modulating the immune response within meningiomas. Ultimately, this approach may improve therapeutic outcomes and quality of life for patients with meningioma by enhancing the effectiveness of existing treatments or by offering new immunotherapeutic options.

**Supplementary Information:**

The online version contains supplementary material available at 10.1186/s13046-025-03419-2.

## Background

Meningiomas are the most common primary intracranial tumours, accounting for over a third of all primary central nervous system (CNS) tumours [1]. Originating from the meningeal cells, they are classified into grade 1 (benign), grade 2 (atypical) and grade 3 (anaplastic/malignant) based on histopathology according to the 2021 WHO Classification of Tumours of the Central Nervous System [2, 3]. While most meningiomas are benign grade 1, some grade 1 exhibit aggressive behaviour, and approximately 20% of cases present as higher-grade tumours with an increased risk of recurrence [4].

Meningiomas are also stratified by molecular characteristics, including genotypes and methylation classes (MCs), to improve prognostic precision. Next-generation sequencing (NGS) has revealed common driver mutations, such as NF2, AKT1, KLF4, TRAF7, SMO, POLR2A [5]. NF2 mutations occur in all grades and *AKT1 E17K* and *KLF4 K409Q* often co-occur with TRAF7 mutations predominantly in grade 1 tumours [6, 7]. Wang et al. recently reviewed the meningioma DNA methylation-based classification systems [6]. Initially, the Heidelberg classifier identified six classes (benign MC ben-1/-2/-3, intermediate MC int-A/-B, and malignant MC mal) [8], while subsequent multi-omic approaches, combining DNA methylation, mRNA expression, and copy number alterations, have yielded additional prognostic categories across various grades and molecular subtypes [9–11]. These molecular subtypes correlate with recurrence risk and clinical outcomes, yet their interplay with the TME remains poorly understood.

Most benign meningiomas can be treated with surgical resection, while inoperable, aggressive or recurrent tumours often require radiotherapy [3, 12]. Effective approved chemotherapy options currently do not exist, and immunotherapy has had mixed success. A phase 2 trial (NCT03279692) of pembrolizumab, a PD-1 inhibitor, showed promising results with prolonged survival in recurrent higher-grade meningiomas [13]. Conversely, the trial (NCT02648997) of nivolumab, another PD-1 inhibitor, failed to improve six-month progression-free survival (PFS-6), although it did achieve durable tumour control [14]. This discrepancy may be attributed to the “cold” TME in meningiomas, highlighting the need to better understand tumour-immune interactions for developing effective combination therapies [15].

To better understand immunotherapy potential, it is important to examine the immune cell composition in the meningioma TME. Tumour-immune interactions are key to tumour progression and therapy response [16]. Meningiomas are unique among CNS tumours as they are not protected by the blood-brain barrier, allowing easier access to peripheral immune cells [15]. TAMs are the most abundant immune cells in meningioma, with M2-like TAMs linked to larger tumours, higher grades and recurrence [17–20]. However, the distribution and role of immune cells, particularly TAMs, across different genotypes and MCs remain unclear. Microglia, the brain’s resident macrophages, play a crucial role in tumour-brain crosstalk, but their involvement in meningiomas is not well understood [21–23]. Tumour-infiltrating lymphocytes, predominantly T cells, are also present in the TME, with lower CD8 + T cell counts and higher regulatory T cell levels correlating with an immunosuppressive TME that may promote tumour growth and immune evasion [24, 25]. Despite these insights, the spatial distribution of immune cells across molecular subtypes and their function remain unresolved.

To gain deeper insights into the TME in meningiomas, we conducted a comprehensive analysis immune cell proportions across different genotypes, MCs, and tumour grades. Our results revealed benign NF2-mutant and MC ben-1 meningiomas exhibited the higher proportions of M2-like TAMs, especially brain-resident microglia and M2-like microglia, whereas aggressive MC mal tumours showed an increased presence of monocyte-derived macrophages and M2-like macrophages. To investigate the role of M2-like TAMs in meningioma progression, we developed a 3D co-culture model using primary meningioma cells and M2-polarised macrophages from the same patient. In this model, M2-like TAMs significantly promoted tumour cell proliferation and suppressed invasion, implicating IL-6-mediated signalling in driving these tumour behaviours. This 3D model offers a valuable platform for studying TAM functions and immune interactions, with potential for developing TAM-targeted immunotherapy strategies.

In summary, this study reveals the heterogeneous TME in meningiomas subtypes, highlighting significant differences in M2-like TAM composition across distinct genotypes and MCs. We also show that M2-like TAMs promote tumour growth while limiting invasion in a 3D co-culture model. These findings offer valuable insights for developing targeted treatments, potentially improving therapeutic precision and efficacy across meningioma subtypes.

## Methods

### Clinical samples

Meningioma tissue and blood samples were collected with informed patient consent and stored according to the Human Tissue Act (2004). Anonymised meningioma (MN) samples from the Plymouth Brain Tumour Biobank (REC No: 24/SC/0278; IRAS No: 345630) were collected under ethical approvals from University Hospitals Plymouth NHS Trust and North Bristol NHS Trust, and the study was performed in accordance with the ethical standards as laid down in the 1964 Declaration of Helsinki and its later amendments. Histopathological confirmation of tumour types and grades was performed by Department of Neuropathology at each site. Ten additional samples were supplied by the Department of Neuropathology Biobank, Heidelberg University Hospital, Germany (S-318/2022). Data for all samples used in this study are detailed in Supplementary Table 1.

### Tissue collection and processing

Fresh tumour samples were collected during surgery and transported in Hibernate™ A medium (Thermo Fisher Scientific), with 100 U/mL penicillin/streptomycin (P/S) (Thermo Fisher Scientific) and 1% Amphotericin B (Merck)). Upon arrival, samples were washed in DPBS (Thermo Fisher Scientific) and placed in a sterile 10 cm dish with appropriate culture medium: MN1 (grade 1: DMEM (Thermo Fisher Scientific), 10% FBS (Merck), 100 U/mL P/S (Thermo Fisher Scientific), 1% GlutaMAX™ (Thermo Fisher Scientific)) or MN2 (grade 2: DMEM/F-12 (Thermo Fisher Scientific), 20% FBS, 100 U/mL P/S, 1% GlutaMAX™) [26]. Tissue was dissected with some pieces snap-frozen for DNA/RNA extraction. Remaining tissue was digested in 0.05% collagenase type 3 at 37℃ for 30 min, then minced and incubated in red blood cell lysis buffer (Thermo Fisher Scientific). After filtration, the single-cell suspension was centrifuged, seeded in culture plates or flasks, and incubated at 37 °C with 5% CO_2_. Fresh medium was replaced every three days.

### Primary cell culture and passaging

Meningioma primary cells from the initial tumour sample (passage 0, P0) were cultured in six-well plates. For passaging, cells were detached using 0.25% Trypsin-EDTA (Thermo Fisher Scientific), resuspended in grade-specific culture medium, and designated as passage 1 (P1). Subsequent passages (P1 to P6) were seeded in fresh plates and maintained at 37 °C in humidified atmosphere with 5% CO_2_. Fresh medium was replaced every three days. These monolayer cultures are referred as “2D” in this study.

### Spheroid culture

The methodology was optimised by our research group [26]. Briefly, P0 primary cells were resuspended in complete spheroid growth medium (GFS) consisting of DMEM/F-12 and Neurobasal (Thermo Fisher Scientific) (1:1), 5% FBS, 1% B27 supplement (Thermo Fisher Scientific), 1% N2 supplement (Thermo Fisher Scientific), 20 ng/mL recombinant human epidermal growth factor (EGF) protein (Bio-Techne), 20 ng/mL recombinant human basic fibroblast growth factor (bFGF) protein (Bio-Techne), 100 U/mL P/S, 1% GlutaMAX™, and 1% non-essential amino acids (NEAA) (Thermo Fisher Scientific). Cells were plated in ultra-low attachment (ULA) 96-well plates (Greiner Bio-One) at 3,000 cells per well, centrifuged at 1,500 rpm for 15 min, and incubated on an orbital shaker at 65 rpm at 37 °C with 5% CO_2_ for three days to allow spheroid formation. Spheroids (3D) were exclusively generated from monolayer (2D) at one passage earlier, for example, P0 2D formed P1 3D after passaging. After three days, intact spheroids were collected for further treatment, including formalin fixation, protein or RNA extraction.

### Driver mutation analysis

Genomic DNA was extracted from frozen meningioma tissues using the DNeasy^®^ Blood & Tissue kit (QIAGEN) and assessed for concentration and quality with a Nanodrop 2000 (Thermo Fisher Scientific, RRID: SCR_018042). DNA was sequenced by the Southwest Genomic Laboratory Hub using the Illumina TruSight Oncology 500 (TSO500) panel [27]. Raw data were analysed with TruSight Oncology 500 v2.2 Local App. Driver mutations were identified and annotated using Cancer Genome Interpreter (CGI) and ANNOVAR, with filtering criteria: gene coverage (50 ×) ≥ 90%, FATHMM_MKL score ≥ 0.5 [28], QUALITY score ≥ 30 [29], variant allele frequency (VAF) ≥ 0.05 [30], minor allele frequency (MAF) ≤ 0.05 [27], and read depth ≥ 100 [31, 32].

### KASP™ genotyping

DNA was extracted using the DNeasy^®^ Blood & Tissue Kit, and concentrations were measured using Nanodrop 2000. Genotyping of *AKT1 E17K* and *KLF4 K409Q* based on bi-allelic single nucleotide polymorphisms (SNPs) was performed using Kompetitive Allele Specific PCR (KASP™) (LGC Genomics Ltd.) on Light Cycler 480 II (Roche). Reactions were carried out in a 10 µL volume with genomic DNA (40 ng/µL), 2 × KASP low ROX Master mix and allele-specific primers (Additional file 2). Synthetic gene fragments with known heterozygous or homozygous mutations (gBlocks, Integrated DNA Technologies) served as positive control [33].

### DNA methylation analysis

DNA was extracted from formalin-fixed paraffin-embedded (FFPE) tumour sections at the Southwest Genomic Hub and the Department of Neuropathology Heidelberg, respectively, and analysed using the Illumina Methylation EPIC Beadchips, covering approximately 850,000 CpG sites [8]. Data were processed on the Heidelberg Molecular Neuropathology platform (https://www.molecularneuropathology.org/mnp/) with the Heidelberg Brain Tumour Classifier (v2.4 for MN201, MN255, and MN315, v12.5 for MN428, MN251, MN566, MN573, MN580, and MN582, v12.8 for other samples) [34] to assign MCs with scores ≥ 0.9 and subclasses with scores ≥ 0.5. Copy number variation (CNV) analysis identified 22q deletions/NF2 mutations and classified *MGMT* promoter status as unmethylated (MGMT-STP27) [35]. This data was integrated with histology for comprehensive MC classification. All MC and CNV data for the analysed samples are present in Supplementary Table 1. Some samples with CNVs lacked an MC subclass due to insufficient scores.

### Multiplex immunohistochemistry (mIHC)

Tissues and spheroids were formalin-fixed, dehydrated, paraffin embedded, and sectioned (4 μm). Sections were dewaxed, rehydrated, and stained with haematoxylin and eosin (H&E) for tumour verification. Tissue sections were treated using the Opal system (Akoya, Opal manual IHC kit) with antigen retrieval in AR6 buffer. After blocking, sections were incubated overnight with the first primary antibody at 4 °C, followed by Opal polymer Ms + Rb HRP and Opal fluorophore. The stripping step removed unbound residues for additional target detection. DAPI was applied for nuclear staining, and slides were mounted with VectaShield (VWR) before coverslipping. The composition and staining order of all primary antibodies and corresponding Opal fluorophores is listed in Supplementary Table 3. Markers included CD68 for macrophages, CD163 for M2-like subtypes, P2RY12 and TMEM119 both for microglia [36–38], and CD3 for T cells. We examined CD68 + total TAMs, CD163 + M2-like TAMs, CD68 + P2RY12 - TMEM119 - macrophages, and CD68 + P2RY12 + TMEM119 - or CD68 + P2RY12 - TMEM119 + or CD68 + P2RY12 + TMEM119 + microglia. We also identified CD163 + macrophages and microglia as M2-like macrophages and microglia, along with CD3 + T cells. Fluorescent images were acquired using a Leica Stellaris confocal microscope (RRID: SCR_024664), processed with Leica Application Suite X (LAS X, RRID: SCR_013673), and quantified in QuPath (https://qupath.github.io/, RRID: SCR_018257). Whole-tissue images of nine genotyped samples were scanned with the PhenoImager™ HT system (RRID: SCR_023772) for further analysis using Phenochart 1.1.0 (Akoya, RRID: SCR_019156).

### Bulk RNA sequencing (RNA-seq) and data analysis

Total RNA was extracted from matched tissue, 2D and 3D derived from the same patient using the Direct-zol™ RNA Miniprep kit (Zymo Research). RNA concentration and quality were assessed by Nanodrop 2000, then sent to Novogene for transcriptomic analysis. After quality control by Agilent 5400, messenger RNA (mRNA) was purified and libraries prepared for RNA-seq using an Illumina NovaSeq 6000 (150 bp paired-end reads). Raw data was processed, mapped to the human reference genome GRCh38 using Hisat2 v2.0.5, and gene expression levels were estimated as Fragments Per Kilobase of transcript sequence per Millions mapped reads (FPKM) using StringTie (v1.3.3b) and FeatureCounts (v1.5.0-p3). Immune cell proportions were analysed using CIBERSORTx (https://cibersortx.stanford.edu/, RRID: SCR_016955) [39]. This software compares FPKM values to the LM22 reference signature matrix using a deconvolution algorithm, which differentiates human immune cell populations.

### Western blotting

Total protein was extracted from cells and spheroids using RIPA buffer (Thermo Fisher Scientific) with Halt™ Protease and Phosphatase Inhibitor cocktail (Thermo Fisher Scientific). Spheroids underwent three freeze-thaw cycles and two sonication cycles for complete lysis. Lysates were centrifuged, and the supernatant was stored at -80 °C. Western blotting was performed as previously described, with membranes incubated with primary and secondary antibodies (listed in Supplementary Table 3) [40]. Chemiluminescence (Thermo Fisher Scientific) detected bands, and ImageJ software (RRID: SCR_003070) was used for densitometric quantification.

### Peripheral blood mononuclear cell (PBMC) isolation

Whole blood was collected in EDTA tubes from three independent grade 1, NF2-mutant patients who provided the corresponding tissue samples. PBMCs were isolated by density gradient centrifugation using Histopaque^®^-1077 (Sigma) in Leucosep tubes (Greiner Bio-One) [41]. After centrifugation, the cell fraction was transferred, washed with PBS, and either used for differentiation or cryopreserved in liquid nitrogen.

### Differentiation of macrophages and M2 polarisation

PBMCs were plated at 8 × 10^5^ cells/well in a 24-well plate using RPMI-1640 medium (Thermo Fisher Scientific) with 10% heat-inactivated FBS and 100 U/mL P/S. The next day, the medium was replaced with macrophage differentiation medium containing RPMI-1640 Glutamax (Thermo Fisher Scientific), 10% heat-inactivated FBS, 100 U/mL P/S and 25 ng/mL macrophage-colony stimulating factor (M-CSF) (Peprotech). On day 5, the medium was switched to promote M2 polarisation, containing 50 ng/mL M-CSF, 40 ng/mL IL-4 (Peprotech) and 40 ng/mL IL-13 (Peprotech) [42]. Cells were collected with Accutase (Merck) on day 7 for further experiments. M2 polarisation was confirmed by immunocytochemistry showing positive CD68 and CD163 expression and negative TMEM119 expression.

### Establishment of a 3D co-culture model containing tumour cells and M2-polarised macrophages

P4 or P5 tumour cells (3,000 cells per well in 100 µL of GFS) were seeded into ULA 96-well plates on Day − 3 to initiate single spheroid formation. Plates were incubated at 37 °C with 5% CO₂ on an orbital shaker set to 65 rpm to promote uniform spheroid aggregation. On Day 0, each mature spheroid was overlaid with 3,000 matched M2‑polarised macrophages, establishing a 1:1 macrophage-tumour cell ratio. Co‑cultures were then maintained under the same incubation and agitation conditions (65 rpm, 37 °C, 5% CO₂) for the duration of the experiment. Control wells contained spheroids cultured in parallel without the addition of matched M2-polarised macrophages. All subsequent functional assays were performed on three independent biological replicates, each from a different grade 1, NF2-mutant patient, with three technical replicates per condition.

### Double colour cell tracking

Tumour cells were stained with 5 µM CellTrace™ CFSE (Thermo Fisher Scientific) for 20 min at 37 °C, then incubated with complete GFS for 5 min. After centrifugation, cells were resuspended in complete GFS to form spheroids. M2-polarised macrophages were stained with CellTracker™ Orange CMTMR (Thermo Fisher Scientific) for 30 min at 37 °C and added to the spheroids according to the 3D co-culture protocol.

### Immunocytochemistry (ICC)

Differentiated macrophages were fixed in 4% paraformaldehyde (PFA) (Thermo Fisher Scientific) for 10 min at room temperature (RT), permeabilised with 0.1% Triton X-100 (Sigma) for 10 min, and blocked in PBS containing 20% Tween with 1% bovine serum albumin (BSA) and 22.52 mg/mL glycine for 1 h at RT. Primary antibodies were incubated overnight at 4℃, followed by secondary fluorophore-conjugated antibodies and DAPI for 1 h at RT in the dark. Fluorescent images were captured using a Leica IM8 microscope. For spheroid staining, incubation time was extended for better penetration and staining of the 3D structure. Spheroids were fixed in 4% PFA for 30 min, permeabilised in 0.5% Triton X-100 for 1 h, and blocked for 2 h at RT. Primary antibodies were then incubated overnight at 4℃, and secondary antibodies with DAPI were incubated for an additional overnight period at 4℃. Images were acquired using a Leica Stellaris microscope. Antibody details are in Supplementary Table 3.

### Live & dead cells assay

Live/Dead^®^ viability/cytotoxicity assay kit (Thermo Fisher Scientific) was used to assess the viability of the 3D co-culture model. Models were incubated in DPBS with 1 µM Calcein AM (live cells, green) and 4 µM Ethidium homodimer-1 (dead cells, red) for 30 min at 37 °C. Samples were then imaged using a Leica Stellaris microscope.

### 3D cell invasion

Medium was removed carefully to avoid disturbing the spheroids. Matrigel™ Basement Membrane Matrix (Thermo Fisher Scientific) was added to each spheroid (80 µL) and allowed to solidify, after which 100 µL of GFS was added. Spheroid invasion was assessed at 24 and 48 h using a Leica IM8 microscope in bright-field mode, and the invaded area was quantified using ImageJ software.

### RNA isolation and real-time RT-PCR

Total RNA was extracted from the groups of only spheroids, only macrophages or 3D co-cultures using the PureLink™ RNA Micro Kit (Thermo Fisher Scientific). RNA concentration was measured with Nanodrop 2000. For cDNA synthesis, 10 µL RNA was converted using the High Capacity cDNA Reverse Transcription Kit with RNase Inhibitor (Thermo Fisher Scientific). Quantitative real-time PCR (qPCR) was performed with TaqMan™ Fast Advanced Master Mix and TaqMan™ Assays (Thermo Fisher Scientific) on a Light Cycler 480 II (Roche), with each sample analysed in triplicate. Gene expression was calculated using the 2^−ΔΔCt^ method [43]. Probes are listed in Supplementary Table 2.

### cBioportal analysis

Data was downloaded from the publicly available Meningioma cBioportal dataset (https://www.cbioportal.org/study/clinicalData?id=mng_utoronto_2021) [9]. 121 samples with mRNA expression data (Log2 transcripts per million) for *IL6*, *TNF*, *CSF1*, and *TGFB1* were identified. Samples were stratified into top and bottom 50% based on expression levels and compared with tumour recurrence proportion.

### Statistical analysis

Statistical analyses were performed based on the type of comparison. Two-way ANOVA was used for multiple comparisons across subgroups with two or more groups, mixed-effects analysis for matched tissue, 2D, and 3D samples, and one-way ANOVA for comparisons across more than two groups. Unpaired Student’s t-test was used for comparisons between two individual groups, and Fisher’s exact test for cBioportal data. GraphPad Prism 9 (RRID: SCR_002798) was used for statistical charts. Experiments were repeated with samples from different patients, and data are presented as mean ± SD. Statistical significance was set at *p* < 0.05.

## Results

### Molecular classification

Fifteen NF2-mutant cases, confirmed by NGS and negative phospho-Merlin expression via Western blotting, were included, along with ten *AKT1 E17K* and nine *KLF4 K409Q*, validated by NGS after KASP™ genotyping. Phospho-Merlin expression was observed in non-NF2 meningiomas. MCs were assigned based on analysis from the Southwest NHS Genomic Laboratory Hub or the Department of Neuropathology, Heidelberg. In total, seven MC ben-1, six MC ben-2, four MC ben-3, six MC int-A and eight MC mal samples were analysed, alongside 35 grade 1, 10 grade 2 and four grade 3 samples. Detailed sample information is presented in Supplementary Table 1.

### TME landscape slightly varied in different grades of meningioma tissue

**Fig. 1:**
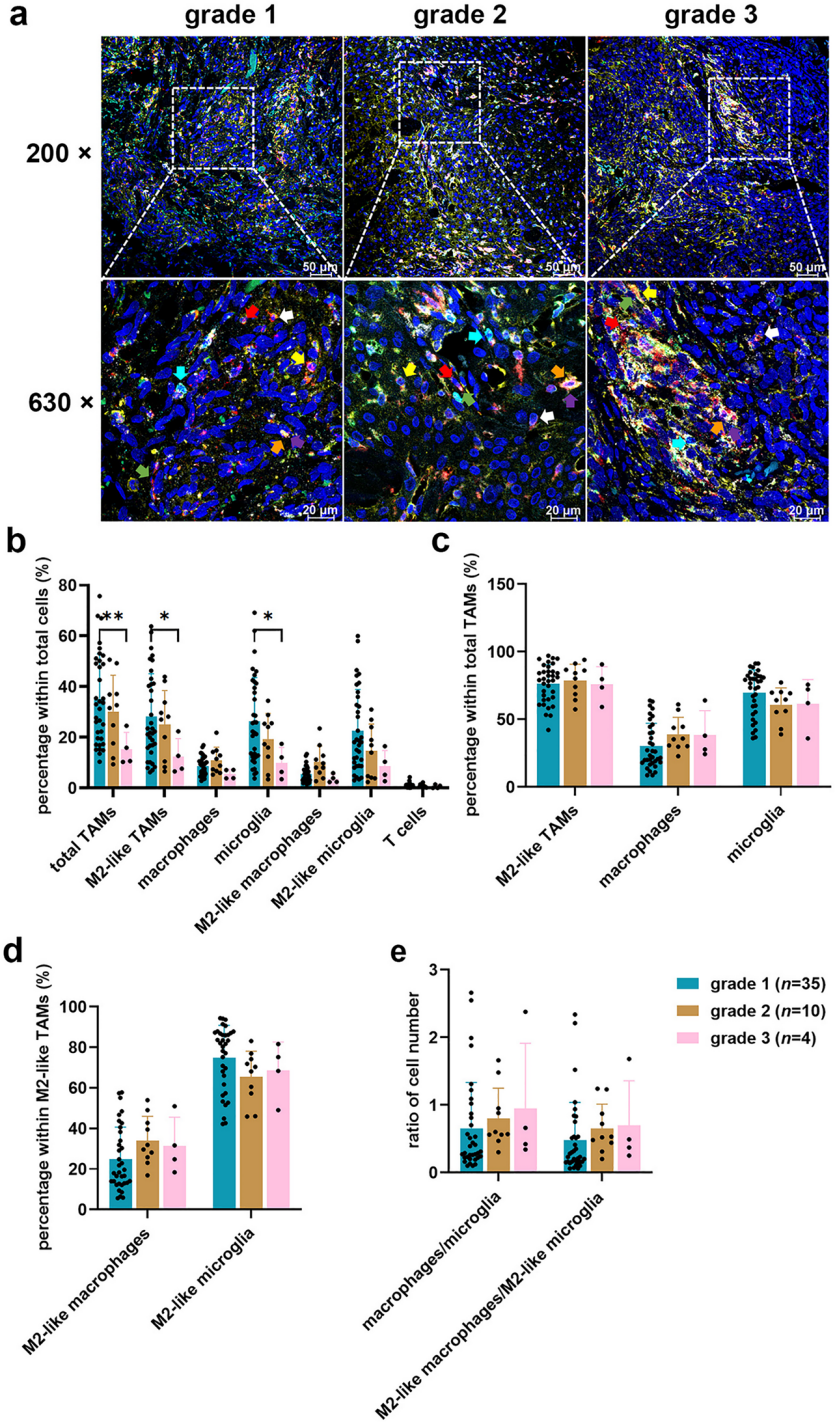
TME landscape in meningioma tissue across different WHO grades. (**a**) Representative images displaying the expression and spatial distribution of five immune markers in grade 1 (*n* = 35), grade 2 (*n* = 10) and grade 3 (*n* = 4). FFPE tissue sections were stained using mIHC for CD68 (pan macrophages, red), CD163 (M2 macrophages, green), TMEM119 and P2RY12 (microglia, white and yellow, respectively), and CD3 (T cells, cyan). The images were captured at 200× and 630× magnification using Leica Stellaris. Coloured arrows indicate specific cell types: red arrow: total TAMs (CD68 +), yellow arrow: M2-like TAMs (CD68 + CD163 +), green arrow: macrophages (CD68 + TMEM119 - P2RY12 -), orange arrow: microglia (CD68 + TMEM119 + or CD68 + P2RY12 + or CD68 + TMEM119 + P2RY12 +), white arrow: M2-like macrophages (CD163 + macrophages), purple arrow: M2-like microglia (CD163 + microglia), and cyan arrow: T cells (CD3 +). (**b**) Quantification of the proportion of each immune cell type in total cells. (**c**) Quantification of the percentage of TAM subtypes in total TAMs cell population. (**d**) Percentage of M2-like TAM subtypes within M2-like TAMs. (**e**) Ratio of cell number among different subtypes of TAMs and M2-like TAMs. Analysis of cell counts was conducted using QuPath software based on the distinct marker expressions. Statistical analyses were performed using two-way ANOVA with Tukey’s multiple comparisons test. *p < 0.05, **p < 0.01

We performed mIHC on meningioma tissue across different WHO grades to assess immune cell composition. Differences in immune infiltration were observed, grade 1 samples exhibited higher total TAMs, M2-like TAMs, and microglia than grade 3 (Fig. 1a, b). However, no significant differences were found in the proportions of M2-like TAMs, macrophages, microglia, M2-like macrophages and microglia or the macrophage-to-microglia ratio, including M2-like subtypes (Fig. 1c-e). Bulk RNA-seq and CIBERSORTx deconvolution confirmed similar immune cell proportions between grade 1 and 2, with no significant differences in major immune cell types (excluding T cells), and total macrophages (Supplementary Fig. 1a, b). Among T cell subtypes, CD4 memory resting T cells were predominant, slightly higher in grade 2 (Supplementary Fig. 1c). M2 macrophages were more prevalent than M1 in both grades (Supplementary Fig. 1d). Immune scores showed no significant difference between grade 1 and 2 reflecting similar immune infiltration (Supplementary Fig. 1e). Overall, immune infiltration showed minimal variation across WHO grades in meningiomas.

### TME landscape varied in different genotypes of meningioma tissue

**Fig. 2:**
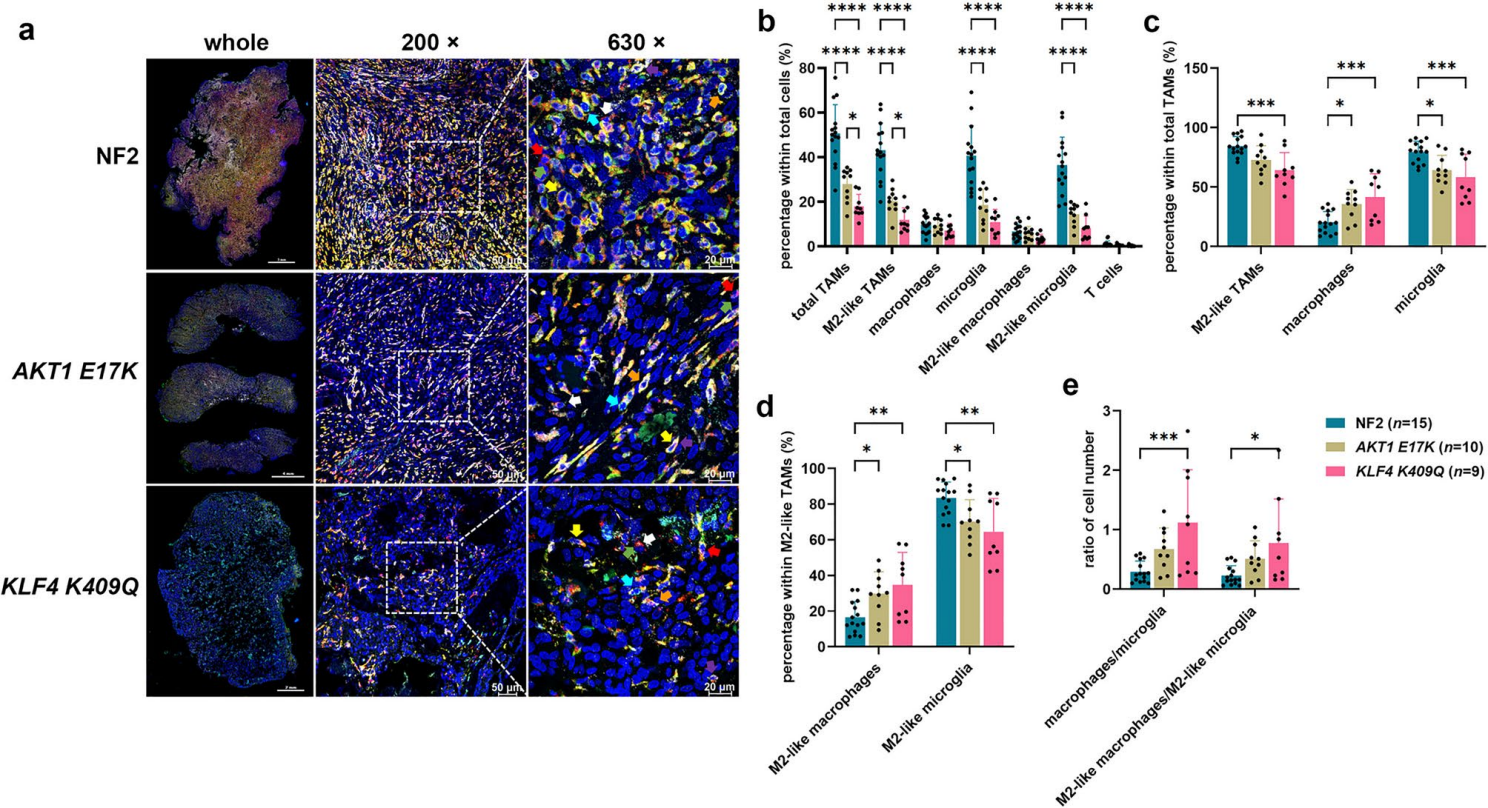
TME landscape in meningioma tissue across different genotypes. (**a**) Representative images illustrating the expression and spatial distribution of five specific immune markers in NF2 (*n* = 15), *AKT1 E17K* (*n* = 10) and *KLF4 K409Q* (*n* = 9). FFPE tissue sections were processed using the same mIHC protocol as Fig. 1a. Whole-tissue images were captured using PhenoImager™ HT system (Akoya). Additional high-magnification images at 200× and 630× were captured using Leica Stellaris. Coloured arrows indicate specific immune cell types: red arrow: total TAMs (CD68 +), yellow arrow: M2-like TAMs (CD68 + CD163 +), green arrow: macrophages (CD68 + TMEM119 - P2RY12 -), orange arrow: microglia (CD68 + TMEM119 + or CD68 + P2RY12 + or CD68 + TMEM119 + P2RY12 +), white arrow: M2-like macrophages (CD163 + macrophages), purple arrow: M2-like microglia (CD163 + microglia), and cyan arrow: T cells (CD3 +). (**b**) Quantification of each immune cell type in the total cell population. (**c**) Percentage of TAM subtypes within total TAMs. (**d**) Quantification of M2-like TAM subtypes within M2-like TAMs. (**e**) Ratio of cell number between different subtypes of TAM and M2-like TAMs. Analysis of cell counts was conducted using QuPath software, based on the distinct marker expressions. Statistical significance was determined using two-way ANOVA with Tukey’s multiple comparisons test. *p < 0.05, **p < 0.01, ***p < 0.001, ****p < 0.0001

To explore immune infiltration based on molecular characteristics, we performed mIHC on genotyped meningioma tissue. NF2 tumours exhibited more immune cells than *AKT1 E17K* or *KLF4 K409Q*, and *AKT1 E17K* showed slightly higher immune cell counts than *KLF4 K409Q* (Fig. 2a). Quantification revealed significant differences in immune cell populations across genotypes. Although T cell infiltration was limited and did not show significant differences, NF2 had the highest percentages of total TAMs, M2-like TAMs, microglia, and M2-like microglia in total cell population (Fig. 2b). Further analysis revealed that NF2 tumours had the highest proportions of M2-like TAMs and microglia within their respective populations. Conversely, the percentage of macrophages within total TAMs and M2-like macrophages within M2-like TAMs was highest in *KLF4 K409Q* (Fig. 2c, d). Additionally, the macrophage-to-microglia and M2-like macrophage-to-M2-like microglia ratios were lower in NF2 than *KLF4 K409Q*, indicating a greater dominance of microglia and M2-like microglia over macrophages and M2-like macrophages in NF2 (Fig. 2e). RNA-seq confirmed these observations, showing a higher proportion of immune cells in NF2 tumours, particularly macrophages and M2 macrophages (Supplementary Fig. 1f, g). In all three genotypes, M2 macrophages predominated over M1 macrophages (Supplementary Fig. 1i). CD4 memory resting T cells were the most abundant T cell subtype, with no significant differences across genotypes (Supplementary Fig. 1h). Notably, immune scores were significantly higher in NF2 compared to *AKT1 E17K* (Supplementary Fig. 1j). Overall, both mIHC and RNA-seq data consistently demonstrated NF2 exhibited greater TAM infiltration, particularly M2-like TAMs, microglia, and M2-like microglia, compared to *AKT1 E17K* or *KLF4 K409Q*.

### TME landscape varied in different methylation classes of meningioma tissue

**Fig. 3:**
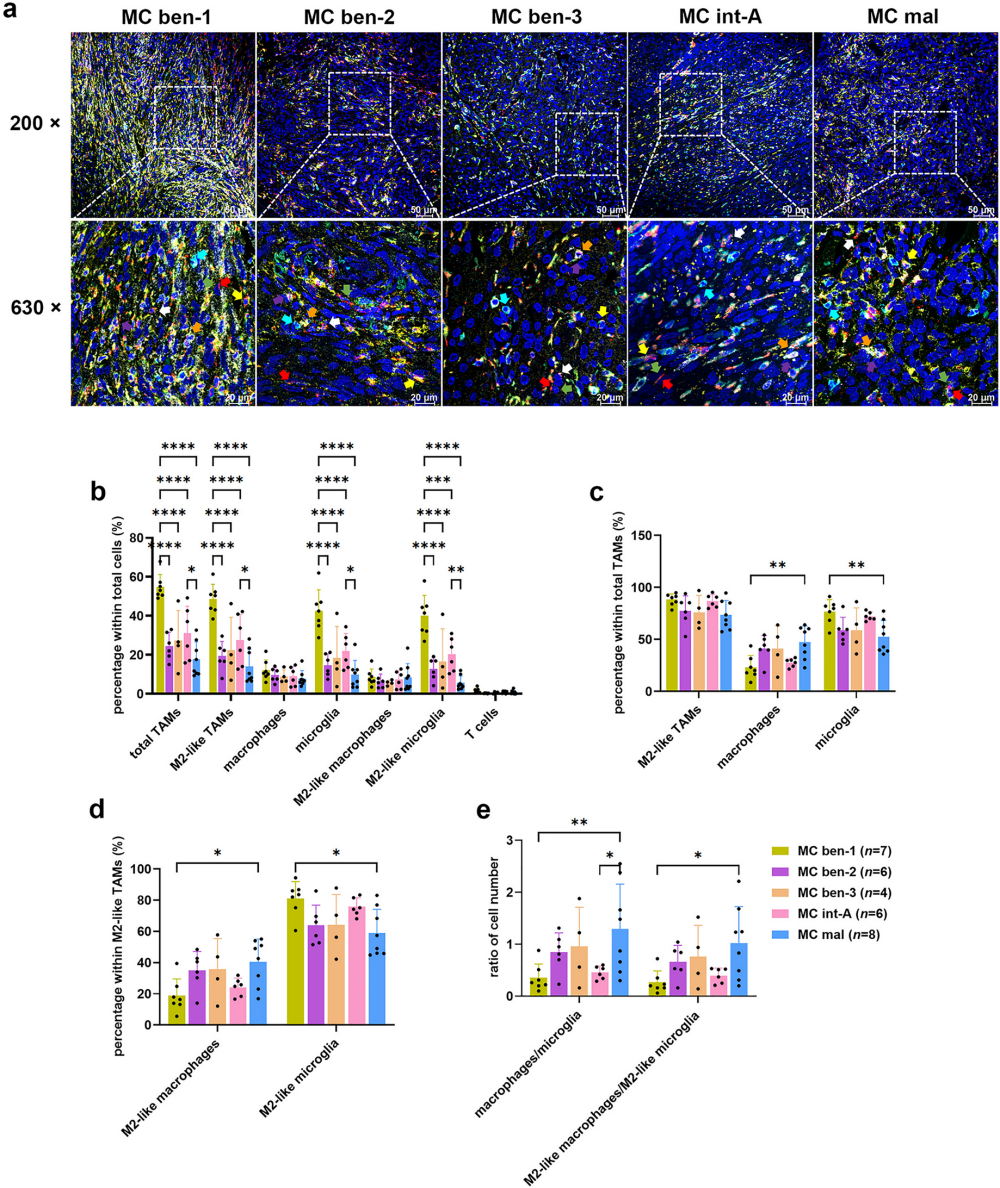
TME landscape in meningioma tissue across different methylation classes (MCs). (**a**) Representative images showing the expression and spatial distribution of five specific immune markers in MC ben-1 (*n* = 7), MC ben-2 (*n* = 6), MC ben-3 (*n* = 4), MC int-A (*n* = 6) and MC mal (*n* = 8). FFPE tissue sections were stained as described in Fig. 1a. High-magnification images at 200× and 630× were captured using Leica Stellaris. Coloured arrows indicate specific immune cell types: red arrow: total TAMs (CD68 +), yellow arrow: M2-like TAMs (CD68 + CD163 +), green arrow: macrophages (CD68 + TMEM119 - P2RY12 -), orange arrow: microglia (CD68 + TMEM119 + or CD68 + P2RY12 + or CD68 + TMEM119 + P2RY12 +), white arrow: M2-like macrophages (CD163 + macrophages), purple arrow: M2-like microglia (CD163 + microglia), and cyan arrow: T cells (CD3 +). (**b**) Quantification of the proportion of each immune cell type in the total cell population. (**c**) Percentage of TAM subtypes within total TAMs. (**d**) Quantification of M2-like TAM subtypes within M2-like TAMs. (**e**) Ratio of cell number between different subtypes of TAM and M2-like TAMs. QuPath was used to analyse the cell number base on distinct marker expressions. Statistical significance was determined using two-way ANOVA with Tukey’s multiple comparisons test. *p < 0.05, **p < 0.01, ***p < 0.001, ****p < 0.0001

We further analysed mIHC data based on MCs of meningiomas. MC ben-1 exhibited the highest immune infiltration, with significantly more total TAMs, M2-like TAMs, microglia, and M2-like microglia in total cell population compared to other groups. MC int-A also showed higher immune cell proportion than MC mal which had the least immune infiltration. T cells expression was low across all MCs, with no significant differences (Fig. 3a, b). Further analysis revealed that MC ben-1 had a higher proportion of microglia within total TAMs and M2-like microglia within M2-like TAMs than MC mal which had more macrophages and M2-like macrophages (Fig. 3c, d). The macrophage-to-microglia ratio was higher in MC mal compared to MC ben-1 and MC int-A, as was M2-like macrophage-to-M2-like microglia ratio (Fig. 3e). When further distinguishing between benign and aggressive MCs, MC ben-1 showed significantly higher percentages of total TAMs, M2-like TAMs, microglia, and M2-like microglia in total cell population compared to the other benign MCs (Supplementary Fig. 2a-d). MC int-A exhibited higher proportions of these immune cells than MC mal, but with a lower proportion of macrophages within total TAMs and M2-like macrophages within M2-like TAMs, and more microglia within total TAMs and M2-like microglia within M2-like TAMs (Supplementary Fig. 2e-g). The macrophage-to-microglia ratio was higher in MC mal than MC int-A (Supplementary Fig. 2h). RNA-seq analysis corroborated macrophages were the predominant immune cell type in all MCs, with higher expression in MC ben-1 (Supplementary Fig. 1k). While M2 macrophages dominated across all MCs, their proportions did not differ significantly between groups (Supplementary Fig. 1l, n). CD4 memory resting T cells were the predominant T cell subtype, with a higher proportion in MC ben-1 (Supplementary Fig. 1m). Immune score reflected higher immune infiltration in MC ben-1 (Supplementary Fig. 1o). Overall, MC ben-1 had the highest TAM infiltration, including M2-like TAMs, microglia, and M2-like microglia, while MC mal had the lowest infiltration and more macrophages and M2-like macrophages.

### 3D maintained the immune feature of parental tissue in different genotypes and methylation classes of meningioma

**Fig. 4:**
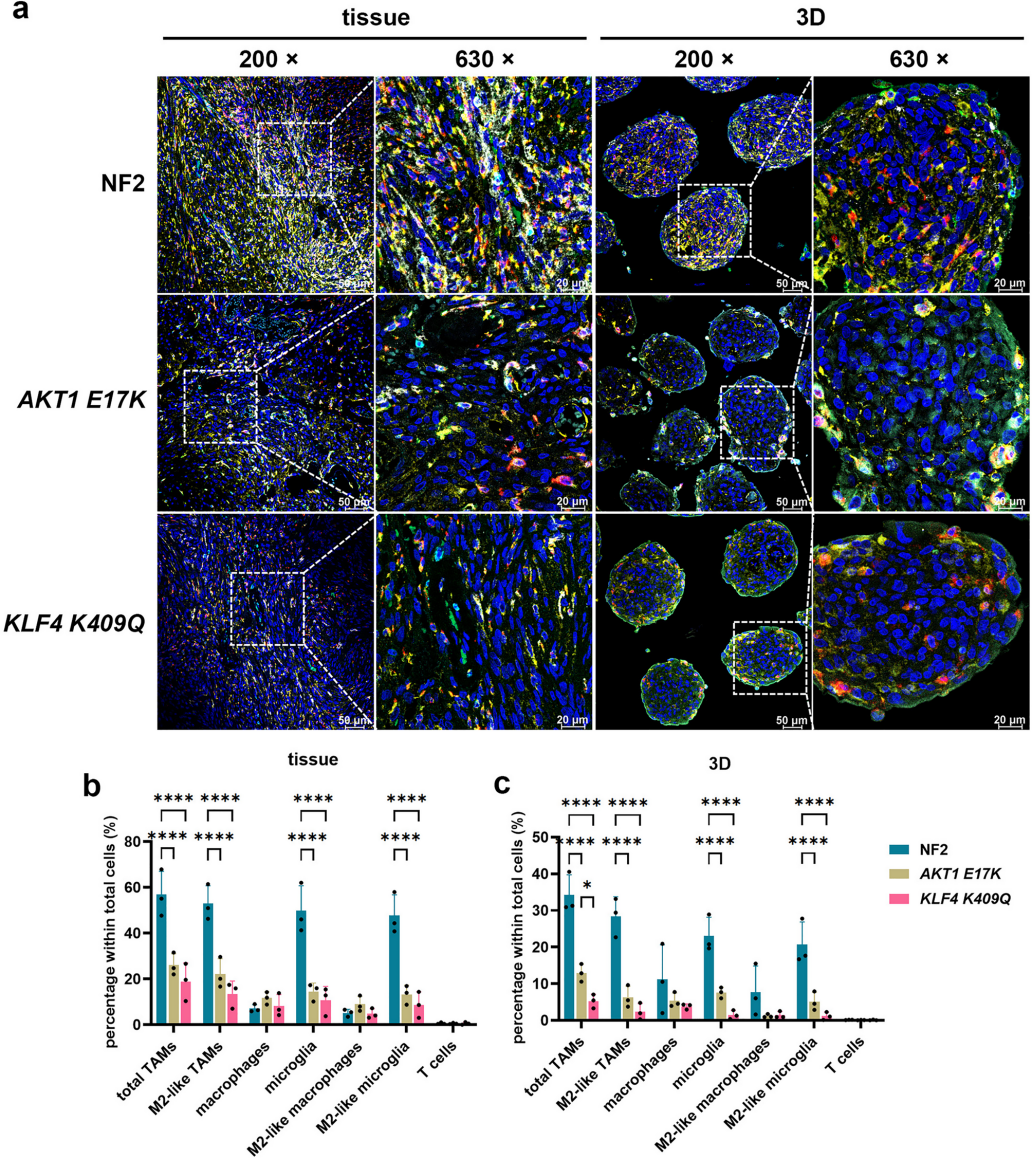
TME landscape in meningioma tissue across different genotypes and matched 3D spheroids models (*n* = 3). (**a**) Representative images displaying the expression and spatial distribution of five specific immune markers in NF2, *AKT1 E17K* and *KLF4 K409Q*. FFPE sections were stained following the same protocol as in Fig. 1a. High-magnification images at 200× and 630× were captured using Leica Stellaris. (**b**) Quantification of the proportion of each immune cell type in the total cell population in parental tissue. (**c**) Proportion of each immune cell type within total cells in matched 3D. Statistical analyses were conducted using two-way ANOVA with Tukey’s multiple comparisons test. *p < 0.05, ****p < 0.0001

To validate an in vitro model for investigating immune cell roles in meningioma, we first compared our recently established 3D meningioma cultures [26] with corresponding parental tissue from the same patients. mIHC on FFPE slides revealed higher immune infiltration in NF2 for both tissue and spheroids (Fig. 4a), with similar immune cell proportions between the two sample types (Fig. 4b, c). Although the 3D model exhibited a lower proportion of immune cells compared to the tissue (likely due to cell loss during spheroid preparation) (Supplementary Fig. 3a-c), there were no significant differences in the proportions of M2-like TAMs or other immune cell subgroups between tissue and 3D models across genotypes (Supplementary Fig. 3d, g, i). However, for *AKT1 E17K* and *KLF4 K409Q*, slight but significant differences were observed M2-like TAM proportions within total TAMs and other TAM subgroups between the tissue and 3D model (Supplementary Fig. 3e, f, h). These differences may be attributed to lower immune infiltration in the parental tissue before the immune cell loss during spheroid preparation. To corroborate these findings, we analysed RNA-seq data using CIBERSORTx deconvolution to compare immune cell populations across genotypes and sample types, including also 2D cultures early passages. Compared to 2D, tissue and 3D revealed similar immune profiles, including significant differences in total and polarised M2 macrophages between NF2 and the other genotypes (Supplementary Fig. 4a-f) being significant in tissue and 3D (Supplementary Fig. 4d-f) The trend of higher M2 macrophages compared to M1 macrophages was consistent across all genotypes in both tissue and 3D samples, with NF2 showing statistical significance (Supplementary Fig. 4g-i).

**Fig. 5:**
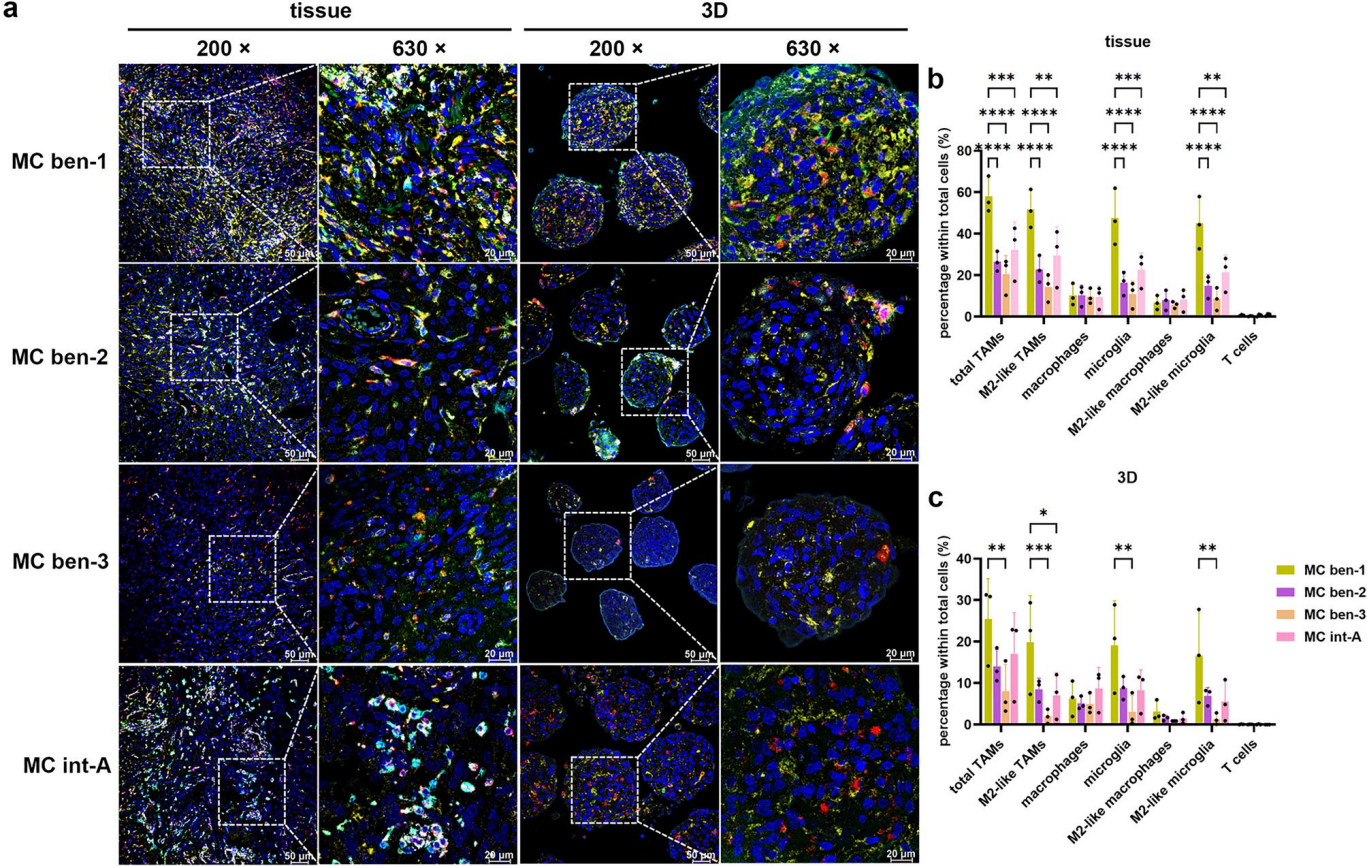
TME landscape in meningioma tissue across different MCs and matched 3D spheroids models (*n* = 3). (**a**) Representative images showing the expression and spatial distribution of five specific immune markers in MC ben-1, MC ben-2, MC ben-3 and MC int-A (without MC mal samples). FFPE sections were stained as described in Fig. 1a. High-magnification images at 200× and 630× were captured using Leica Stellaris. (**b**) Quantification of the proportion of each immune cell type in the total cell population in parental tissue. (**c**) Proportion of each immune cell type within total cells in matched 3D. Statistical analyses were performed using two-way ANOVA with Tukey’s multiple comparisons test. *p < 0.05, **p < 0.01, ***p < 0.001, ****p < 0.0001

Next, we compared immune infiltration in meningiomas classified by MCs in parental tissue and matched spheroids. The immune infiltration pattern in 3D samples mirrored that of parental tissue (Fig. 5a). In both tissue and 3D models, MC ben-1 exhibited higher immune cell percentages compared to other MCs, with significant differences mainly between MC ben-1 and MC ben-3 (Fig. 5b, c). While immune cell proportions were lower in the 3D model compared to parental tissue (Supplementary Fig. 5a-d), no significant differences in TAM subtypes were found between tissue and 3D for MC ben-1 and MC ben-2 (Supplementary Fig. 5e-h). Similarly, no differences in M2-like subtypes within M2-like TAMs was observed for MC ben-1, MC ben-2, and MC int-A (Supplementary Fig. 5i-l). To further investigate, we analysed immune cell composition in parental tissue, matched 2D cultures, and 3D models using CIBERSORTx. Differences in macrophage proportions were observed between MC ben-1 and MC ben-2 or MC ben-3 in the 3D samples, and a similar trend in tissue (Supplementary Fig. 6a-c). Polarised macrophages also exhibited a similar pattern between tissue and 3D samples across the MCs (Supplementary Fig. 6d-i). In conclusion, these results demonstrate that 3D model reasonably recapitulates the immune infiltration patterns of parental tissue in different genotypes and MCs of meningiomas.

### 3D is a better model in vitro than 2D to recapitulate TME of parental tissue

**Fig. 6:**
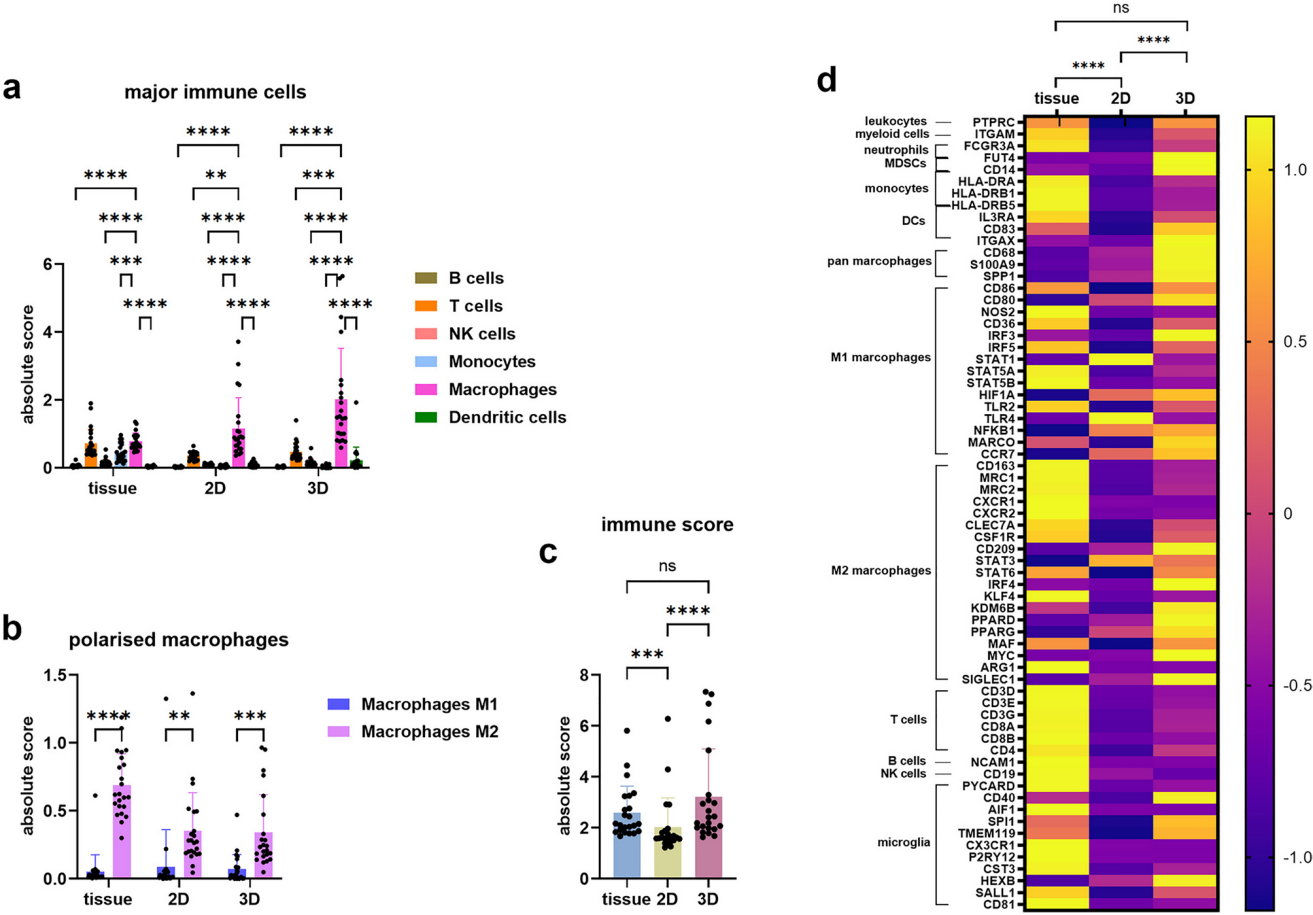
Comparison of immune features in matched tissue, 2D and 3D meningioma samples (*n* = 23). (**a**) Proportion of major immune cells in parental tissue and matched 2D and 3D models. Immune cell representation in the TME was analysed using deconvolution of bulk RNA-seq data with CIBERSORTx. Statistical difference was analysed by mixed-effects analysis with Dunnett’s multiple comparisons test. (**b**) Comparison of the proportion of two polarised macrophage subtypes across different sample types. Statistical difference was analysed using mixed-effects analysis with Sidak’s multiple comparisons test. (**c**) Comparison of the immune score across matched sample types. Statistical analysis was performed using a nonparametric test with Dunn’s multiple comparisons test. (**d**) Heatmap illustrating the expression differences of key immune markers among matched sample types. Centralised data based on RNA-seq FPKM values was visualised to depict the expression patterns of immune markers referenced to Human Immune Cell Marker Guide (https://www.cellsignal.com/pathways/immune-cell-markers-human) and Neuronal and Glial Cell Marker Atlas (https://www.cellsignal.com/pathways/neuronal-and-glial-cell-markers) posters by Cell Signaling Technology. Statistical difference was analysed using a nonparametric test with Dunn’s multiple comparisons test. *p < 0.05, **p < 0.01, ***p < 0.001, ****p < 0.0001

To corroborate if the 3D is a reasonable model to study the role of immune cells in meningiomas, we conducted RNA-seq analysis on matched tissue, 2D, and 3D from the same patient cohort (*n* = 23). Macrophages were the dominant immune cell type across all sample types, with a higher proportion of M2 than M1 macrophages (Fig. 6a, b). Immune score showed significant differences between tissue and 2D, and between 2D and 3D, but no significant difference between tissue and 3D samples, indicating similar immune profiles between tissue and 3D (Fig. 6c). Gene expression analysis of key immune markers revealed consistent patterns between tissue and 3D samples, contrasting with 2D cultures. Markers like CD45 (leukocyte marker, coded by *PTPRC*) [44], CD86 (M1 macrophage marker) [45], CSF1R (M2 macrophage marker) [46], and TMEM119 (microglia marker) [47] exhibited similar expression between tissue and 3D, but differed in 2D (Fig. 6d). Variability in the expression of certain immune markers used for mIHC was observed. The expression of CD68 (pan macrophage marker) [48] was lower, and CD163 (M2 macrophage marker) [49] and P2RY12 (microglia marker) [50] were higher in tissue compared to 3D. Overall, these results suggest that 3D model is a more reliable and accurate representation of immune infiltration in meningiomas than 2D cultures.

### M2-polarised macrophages promoted proliferation but inhibited invasion of meningioma cells in the 3D model

**Fig. 7:**
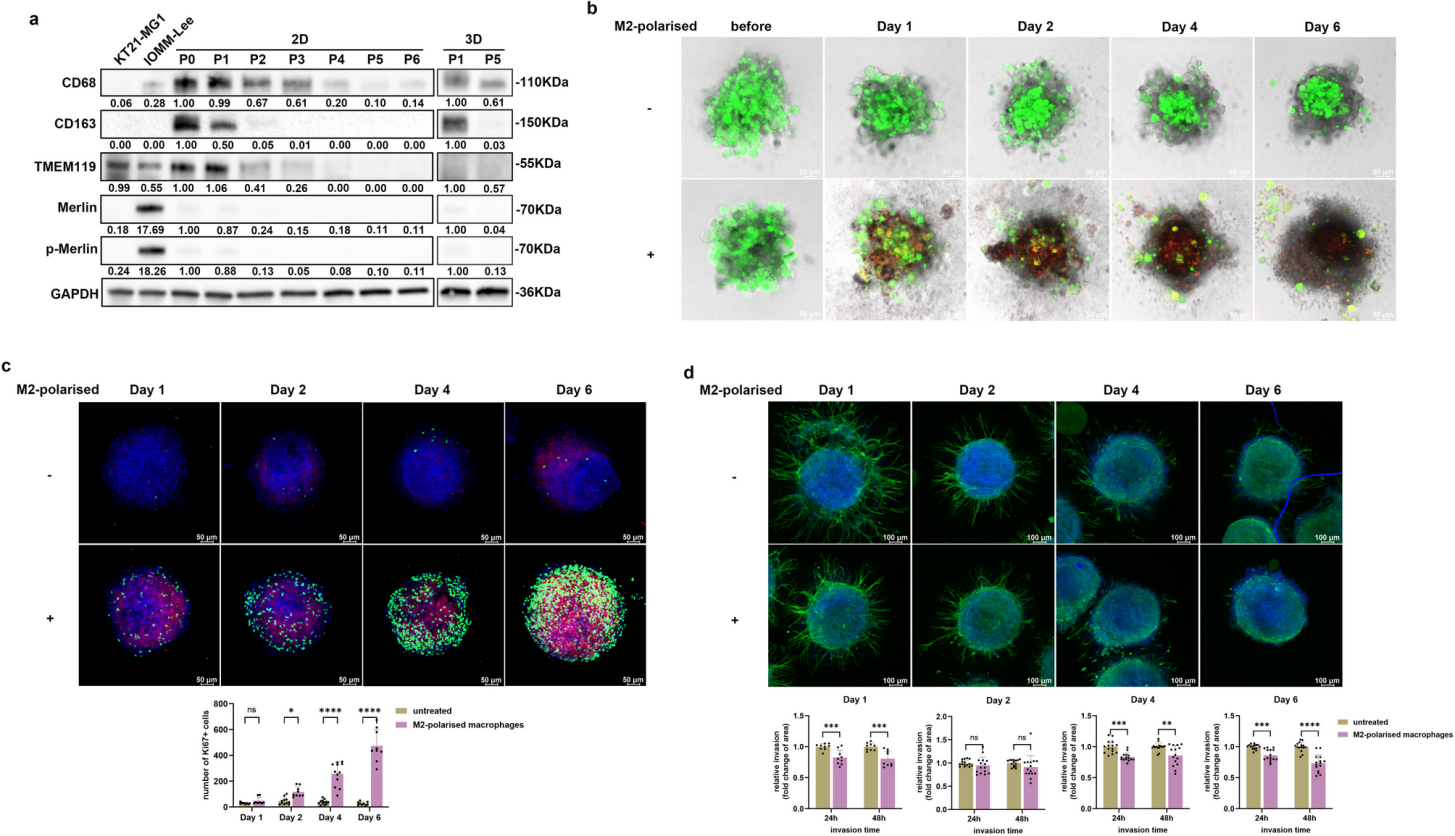
Co-culture models demonstrating the role of M2-like macrophages on tumour cells (*n* = 3). (**a**) Western blotting analysis of immune markers (CD68, CD163 and TMEM119) in 2D and 3D primary cells derived from NF2-negative meningioma samples across different culture passages. KT21-MG1 (a grade 3 meningioma cell line) was used as a Merlin-negative control. IOMM-Lee (another grade 3 meningioma cell line) was the Merlin-positive control. (**b**) Double colour cell tracking illustrating the infiltration of M2-polarised macrophages (red) into tumour cells (green) during the co-culture process over time. Tumour cells were labelled with CellTrace™ CFSE (green), and M2-polarised macrophages with CellTracker™ Orange CMTMR (red). (**c**) Tumour cell proliferation in the co-culture model over time. ICC was used to detect M2-like macrophages marker CD163 (red) and the proliferation marker Ki67 (green), with nuclei stained by DAPI (blue). Statistical difference of Ki67 + cell count was conducted using two-way ANOVA with Sidak’s multiple comparisons test. (**d**) Tumour cell invasion in the co-culture model over time. Matrigel assay was performed to observe the cell protrusions of spheroids over time and stained using ICC with Phalloidin (green) for actin filaments and DAPI (blue) for nuclei. Invasion areas were quantified using ImageJ at 24 h and 48 h and compared between untreated and treated M2-polarised macrophages in 3D models. Statistical difference was analysed using two-way ANOVA with Sidak’s multiple comparisons test. *p < 0.05, **p < 0.01, ***p < 0.001, ****p < 0.0001

Having established the abundance of M2-like TAMs and their increasing number with tumour progression, we developed a 3D co-culture model using M2-polarised macrophages and tumour cells from the same patient to understand the role of M2-like macrophages. First, we determined the optimal passage of primary tumour cells for co-culture by culturing from passage 0 (P0) to passage 6 (P6) and assessing immune marker expression (CD68, CD163, and TMEM119) via western blotting. As expected, CD68 and CD163 expression decreased with increasing passage number [33, 51], and we also observed a reduction in TMEM119 expression (Fig. 7a). The passage with the lowest immune marker expression was selected for the 3D co-culture model to minimise the influence of endogenous TAMs. In the 3D model, M2-polarised macrophages (CD68 + CD163 + TMEM119 -) that had been validated by ICC were co-cultured with tumour cells (Supplementary Fig. 7a). M2-polarised macrophages, labelled with CMTMR (red), infiltrated the tumour cells (labelled with CFSE, green) over time, as demonstrated by double fluorescence staining (Fig. 7b). Co-staining with CD68 and CD163 confirmed M2-polarised macrophage infiltration, compared to spheroids without M2-polarised macrophages (Supplementary Fig. 7b). We next investigated the impact of M2-polarised macrophages on tumour growth. Ki67 (a proliferation marker) and CD163 co-staining showed no co-localisation, indicating that the observed proliferation was due to tumour cells, not M2-polarised macrophages. Tumour cell proliferation was significantly increased in the presence of M2-polarised macrophages over time (Fig. 7c), though cell viability remained unaffected (Supplementary Fig. 7c). To assess tumour invasion, we performed a Matrigel invasion assay. Surprisingly, the invasive protrusions were reduced in the presence of M2-polarised macrophages at most time points (Fig. 7d, Supplementary Fig. 7d), suggesting that M2-polarised macrophages may inhibit tumour cell invasion.

### IL-6 production was elevated in the 3D model with macrophages and correlates with meningioma recurrence

**Fig. 8:**
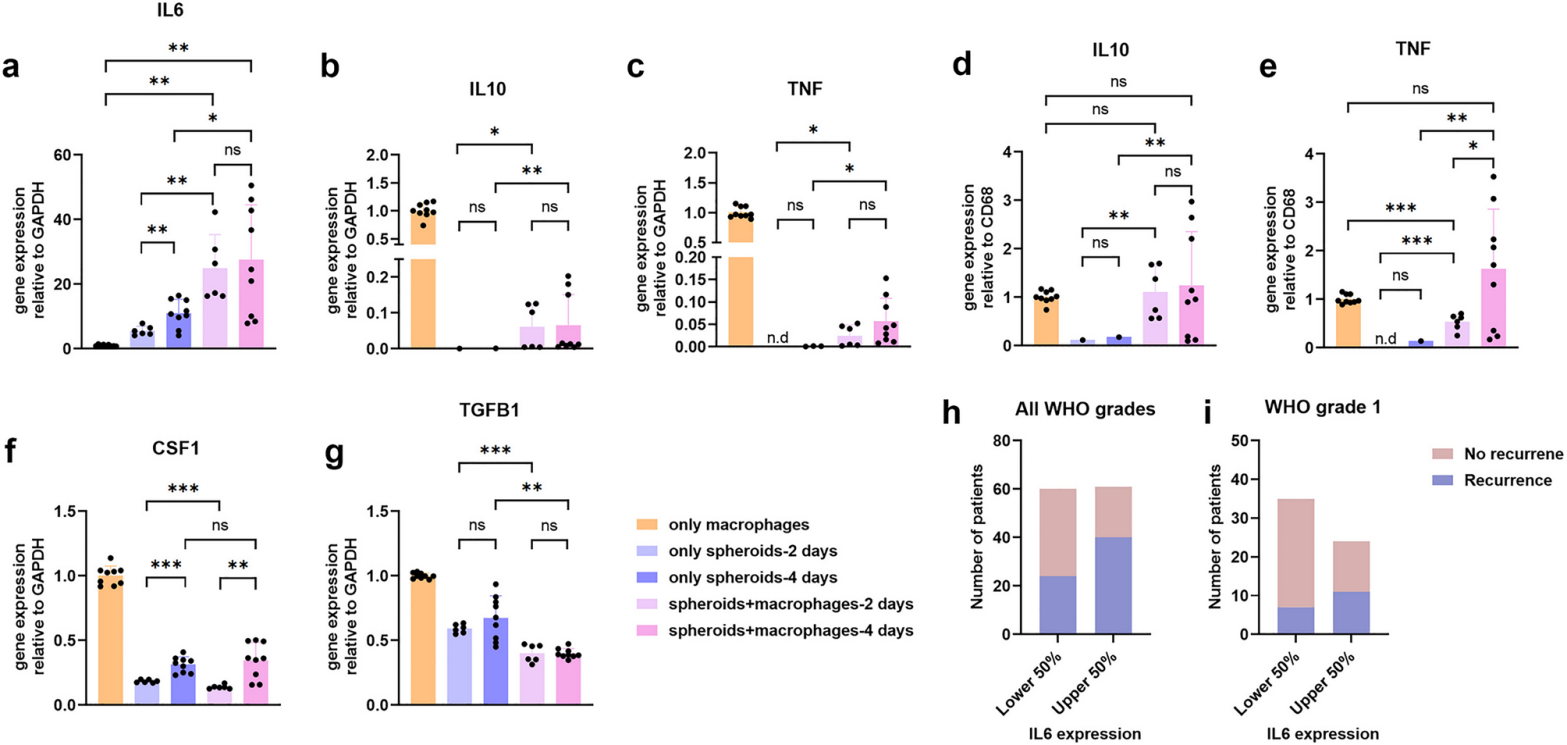
Expression of macrophage-related cytokines in co-culture models (*n* = 3), and correlations between *IL6* expression and recurrence. (**a**-**g**) Cytokine gene expression in cell models. (**a**) *IL6* relative to *GAPDH*, (**b**) *IL10* relative to *GAPDH*, (**c**) *TNF* relative to *GAPDH*, (**d**) *IL10* relative to *CD68*, (**e**) *TNF* relative to *CD68*, (**f**) *CSF1* relative to *GAPDH*, (**g**) *TGFB1* relative to *GAPDH*. Statistical differences were analysed using an unpaired Student’s t-test for normal data or a nonparametric test for non-normal data. *p < 0.05, **p < 0.01, ***p < 0.001, ****p < 0.0001. (**h**-**i**) Correlations between *IL6* expression and tumour recurrence in patients of (**h**) All WHO grades (*p* = 0.0063) and (**i**) WHO grade 1 (*p* = 0.0462). Fisher’s exact test was used for statistical analysis

To explore the interactions between tumour cells and macrophages, we co-cultured spheroids with PBMC-derived macrophages and evaluated macrophage-related cytokine mRNA expression using quantitative RT-PCR. *IL6* expression increased over time in spheroid-only, but was significantly stronger in the co-culture model than macrophage-only or spheroid-only populations, suggesting macrophages enhanced IL-6 production by tumour spheroids (Fig. 8a). *IL10* and *TNF* also showed higher expression in co-cultures compared to spheroids alone (Fig. 8b, c). In contrast to the regulation pattern of *IL6* analysing the cytokine expression relative to *CD68*, a marker expressed only in macrophages, revealed that *IL10* and *TNF* expression stemmed from macrophages within the co-cultures (Fig. 8d, e). *CSF1* expression increased in the spheroid-only group over time but decreased upon co-culture with macrophages (Fig. 8f). Similarly, *TGFB1* expression, initially present in spheroids, decreased after co-culture over time (Fig. 8g). Notably, analysis of the University of Toronto Meningioma dataset from cBioportal revealed a correlation between higher *IL6* mRNA levels and increased tumour recurrence: 65.6% (40/61) of patients in the top half of *IL6* expression develop recurrence, compared with 40.0% (24/60) in the bottom half (*p* = 0.0063, Fig. 8h), especially in grade 1(*p* = 0.0462, Fig. 8I), but not in grade 2 or 3 (Supplementary Fig. 8a, b). No such correlation was observed for *TNF*, *CSF1*, or *TGFB1*, with no significant differences in recurrence rates between high and low expression groups for these cytokines (Supplementary Fig. 8c-e). These results demonstrate that IL-6 plays a significant role in regulating TME in the presence of macrophages and suggest its specific contribution on tumour progression.

## Discussion

TAMs, including macrophages and microglia, are abundant in meningiomas as shown in several studies [38, 52], including ours [33, 51]. This study is the first to show significant differences in both M2-like TAM populations across genotypes and MCs (Figs. 2 and 3). Our findings suggest potential biological mechanisms that may influence meningioma behaviour. Understanding the TME based on genotypes and MCs can provide valuable insights into tumour progression and therapeutic targets [53].

This study examined the TME of meningiomas classified by WHO grades, genotypes and MCs using mIHC and bulk RNA-seq. In general, we found no significant differences in immune infiltration across WHO grades, though grade 1 meningiomas exhibited slightly more total TAMs, M2-like TAMs, and microglia than grade 3. These findings align with previous studies reporting no significant differences in TAM and M2-like TAM infiltration between benign (grade 1) and atypical/anaplastic (grade 2 and 3) meningiomas [54]. However, our results differ from Proctor et al., potentially due to study design variations and limited higher-grade samples [55]. Bulk RNA-seq analysis revealed significantly higher levels of total and CD4 + resting memory T cells in grade 2 meningiomas. Previous research has demonstrated that the density of tumour-infiltrating T cells can serve as a prognostic marker in atypical meningiomas, and CD3 + and CD8 + expression levels play a critical role in the selection of potential T-cell-based immunotherapies [56, 57].

Building on these observations, we examined TAM infiltration patterns across specific genotypes, including NF2, *AKT1 E17K*, and *KLF4 K409Q*, to uncover genotype-dependent differences in the TME. NF2 meningiomas exhibited greater immune cell infiltration, particularly in total TAMs, M2-like TAMs, microglia, and M2-like microglia. Notably, NF2 genotypes demonstrated a higher proportion of M2-like TAMs and microglia in total TAMs, as well as M2-like microglia within M2-like TAMs, but fewer macrophages in total TAM population and M2-like macrophages within M2-like TAMs. The macrophage-to-microglia and M2-like macrophage-to-M2-like microglia ratio was significantly lower in NF2, underscoring a distinct TAM profile. This is the first study to illustrate that brain-resident macrophages (microglia) outnumber bone marrow-derived macrophages in meningiomas. Furthermore, bulk RNA-seq analysis corroborated higher proportion of macrophages and M2-like subtypes in NF2, reflecting total TAMs and M2-like TAMs in samples although CIBERSORTx could not distinguish microglia from macrophages due to dataset limitations. These results suggest higher M2-like TAM infiltration, especially M2-like microglia in NF2. Regarding T cell infiltration, total T cells were less abundant than macrophages with no significant differences across genotypes. CD4 + memory resting T cells were the dominant T cell subtype, indicating limited responsiveness to immune checkpoint blockade (ICB) targeting PD-1/PD-L1 in meningiomas [14]. The higher TAM infiltration and limited T cell presence in NF2 suggest a more immunosuppressive TME, which may contribute to worse clinical outcomes [58], consistent with the enrichment of NF2 mutations in higher grades while AKT1 and KLF4 mutations are predominantly associated with lower grades [59].

We examined immune infiltration patterns in meningioma across different MCs and found that MC ben-1 displayed a higher proportion of total TAMs, M2-like TAMs, microglia, and M2-like microglia compared to other MCs, and MC int-A higher than MC mal. This agrees with previous literature on the immune-enriched features of MC ben-1 [6]. For the first time we have shown that MC ben-1 had a significantly higher percentage of microglia in total TAM population and M2-like microglia within M2-like TAMs compared to MC mal, but a lower proportion of macrophages within total TAMs and M2-like macrophages within M2-like TAMs. The macrophage-to-microglia ratio was higher in MC mal than MC ben-1 and MC int-A, while the M2-like macrophage-to-M2-like microglia ratio only showed difference between MC ben-1 and MC mal. Using a simple IHC method, we suggest that MC ben-1 can be distinguished from MC mal by a higher infiltration of microglia and M2-like microglia and lower proportions of macrophages and M2-like macrophages, which could aid clinical stratification. MC int-A demonstrated greater TAM and subgroup infiltration than MC mal, with more microglia within total TAMs and M2-like microglia within M2-like TAMs but fewer macrophages and M2-like macrophages. This indicates that more aggressive meningiomas have lower immune infiltration or immune cell proportion, with more macrophages rather than microglia, contrasting with benign MCs and genotypes. These findings highlight the importance of distinguishing between benign and malignant MCs when analysing TAM subgroups in meningiomas. Overall, our data show low-grade meningiomas, especially those with NF2 genotype or MC ben-1, exhibit higher TAM infiltration, characterised by more microglia and fewer macrophages. Conversely, higher-grade meningiomas, particularly MC mal, had a TME with a higher proportion of macrophages than microglia. We also hypothesize that microglia may recruit macrophages in aggressive meningiomas, where M2-like macrophages may contribute to tumour progression [60]. However, while P2RY12 and TMEM119 are widely employed as microglia markers, emerging evidence suggests that monocyte derived macrophages can express these markers under certain environmental conditions [61]. Therefore, identification of microglia based solely on P2RY12 and TMEM119 should be interpreted with caution. Complementary approaches, such as incorporating additional microglial markers or utilising single cell transcriptomics, are recommended to more accurately distinguish between microglia and infiltrating monocyte derived macrophages.

Our study using patient-derived 3D spheroid models showed that these spheroids closely mirrored the TME of meningiomas across various genotypes and MCs, resembling their parental tissue (Figs. 4 and 5). Compared to 2D cultures, the 3D model better preserved the immune cell features, making it a more physiologically relevant system for studying tumour-immune interactions. In the 3D co-culture with M2-polarised macrophages from the same patient, we observed that M2-polarised macrophages significantly promoted tumour cell proliferation over time. This suggests that M2-polarised macrophage-stimulated proliferation could explain the decreased TAM proportion during tumour progression as tumour growth outpaces macrophage recruitment. Interestingly, M2-polarised macrophages stimulated tumour cells exhibited reduced invasion, which may link cell invasion to cell cycle regulation, as basement membrane invasion might require cell cycle arrest [62]. In our model, continuous cell cycle activation in the presence of M2-polarised macrophages might inhibit invasiveness. However, since we used grade 1 NF2-mutant meningioma cells, further investigation across other classifications is necessary to fully understand the role of M2-like macrophages in tumour progression.

Our investigation into tumour-immune cell interactions revealed significant changes in macrophage-related cytokine expression in 3D co-culture models. In the presence of macrophages, 3D co-cultures exhibited elevated expression of *IL6*, *IL10*, and *TNF*. IL-6, known for promoting tumour progression through inflammation pathway [63, 64], emerged as a potential therapeutic target for meningiomas. Elevated IL-6 levels in spheroids within the co-culture models, especially in the presence of macrophages, suggest that TAMs may enhance IL-6 secretion by tumour cells to drive tumour progression. cBioportal data confirmed high *IL6* expression in meningioma patients correlated with tumour recurrence, unlike other macrophage-related cytokines. These findings strengthen IL-6’s role in tumour growth with non-proinflammatory mechanisms [65], consistent with our co-culture results. Meanwhile, IL-10 and TNF-α increased only in co-cultures, highlighting a different regulatory pattern from IL-6. Overall, IL-6 may serve as a specific target to modulate the TME and improve meningioma treatment outcomes.

While our study provides valuable insights into meningioma subtyping and the role of TAMs, several limitations warrant considerations. The modest cohort of patient-derived samples and corresponding 3D co‐culture models, constrained by practical and ethical challenges in sample acquisition, limits statistical power and may affect the generalisability of our findings, particularly for rare or aggressive meningioma subtypes. Future studies should incorporate larger, multi-centre cohorts to validate these observations.

In addition to the inter-patient variability, intra-tumoral heterogeneity presents additional challenges, as distinct regions within a single tumour can harbour unique genetic, epigenetic, and microenvironmental characteristics [66]. To address these complexities, future research should integrate single‑cell and spatial transcriptomic approaches to achieve a more nuanced understanding of tumour-immune interactions and improve meningioma stratification.

Our 3D spheroid co-culture system, while offering advantages over 2D monolayers, remains a simplified representation of the TME. It lacks key components, such as endothelial cells, fibroblasts, and pericytes, which are critical for immune cell recruitment, angiogenesis, and ECM remodelling [67]. The absence of a functional vascular network limits the formation of physiologic gradients of oxygen, nutrients, and metabolites, and precludes the study of paracrine signalling loops between tumour cells, stromal fibroblasts, and immune cells [68]. Consequently, although spheroids better mimic in vivo architecture than 2D cultures, they do not fully capture the structural complexity and multicellular crosstalk of native tumours. Future studies should integrate heterotypic cultures, including endothelial and stromal cell populations, or adopt vascularised 3D platforms to develop more physiologically relevant and predictive models of meningioma biology.

The traditional binary classification of macrophages into “M1” (pro‑inflammatory) and “M2” (anti‑inflammatory) subtypes is overly simplistic and does not reflect their true plasticity in vivo. Macrophage activation exists along a dynamic continuum, with overlapping transcriptional and functional profiles influenced by various cytokines, metabolic cues, and spatial context within the TME [69]. Future studies should employ high‑dimensional techniques, such as single-cell RNA-seq or multiplexed spatial imaging, to resolve the full spectrum of macrophage phenotypes, identify hybrid and intermediate states, and more accurately delineate their roles in meningioma progression and therapeutic response.

## Conclusions

Our findings suggest that IHC-based assessment of M2-like macrophages and microglia could enhance progression risk scoring for benign meningiomas in routine pathology labs, especially where molecular profiling is limited. Our study also highlights the 3D cell culture model as a reliable platform for studying the meningioma TME, reflecting the TME of parental tissue. After understanding the role of individual TAMs this model offers a valuable tool for exploring driver mutations and tumour-immune interactions, advancing research into targeted therapies and personalised treatments.

## Electronic supplementary material

Below is the link to the electronic supplementary material.

Supplementary Material 1: Additional file 1

Supplementary Material 2: Additional file 2

## Data Availability

No datasets were generated or analysed during the current study.

## Abbreviations

2D: Two-dimensional monolayer
3D: Three-dimensional, spheroids
bFGF: Basic fibroblast growth factor
BSA: Bovine serum albumin
CGI: Cancer Genome Interpreter
CNS: Central nervous system
CNV: Copy number variation
EGF: Epidermal growth factor
FFPE: Formalin-fixed paraffin-embedded
FPKM: Fragments Per Kilobase of transcript sequence per Millions mapped reads
GFS: Spheroid growth medium
H&E: Haematoxylin and eosin
ICB: Immune checkpoint blockade
ICC: Immunocytochemistry
KASP™: Kompetitive Allele Specific PCR
MAF: Minor allele frequency
MC: Methylation class
mIHC: Multiplex immunohistochemistry
mRNA: Messenger RNA
NEAA: Non-essential amino acids
NGS: Next-generation sequencing
PBMC: Peripheral blood mononuclear cell
PFA: Paraformaldehyde
P/S: Penicillin/streptomycin
RNA-seq: RNA sequencing
RT: Room temperature
SNP: Single nucleotide polymorphism
TME: Tumour microenvironment
TAM: Tumour-associated macrophage
ULA: Ultra-low attachment
VAF: Variant allele frequency
WHO: World Health Organisation
