# Supplementary material for: Tumour-associated macrophage infiltration differs in meningioma genotypes, and is important in tumour dynamics
Source: J Exp Clin Cancer Res. 2025 May 27;44:162. doi: 10.1186/s13046-025-03419-2 (PMC12107748; doi:10.1186/s13046-025-03419-2)
Supplement: Supplementary file 1 — Supplementary Material 1: Additional file 1 [file 13046_2025_3419_MOESM1_ESM.pdf]

Supplementary Table 1 Clinical data and applications for patient samples

Patient	Age	Gender	Grade	Genotype	MC	NF2 mutation	CNVs	Applications	
								mIHC	RNA-seq
MN201	61	M	1		int-A		chr 1p, 3p, 6q, 7p, 19p loss	T, 3D	T, 2D, 3D
MN357	56	F	1	NF2		NM_000268:exon2:c.181_182del:p.F61Lfs*24, NM_181830:exon9:c.853_859del:p.T285Pfs*5		T	
MN373	69	F	1	NF2		NM_181830:exon9:c.866delA:p.A290Hfs*2		T	
MN360	62	F	1	NF2		NM_181830:exon8:c.C709T:p.Q237X		T	
MN378	43	F	1	NF2		NM_181830:exon9:c.C864T:p.N288N, NM_181830:exon4:c.274_280del:p.N92Ifs*3		T	
MN397	51	F	1	NF2		NM_181830:exon9:c.805delA:p.T269Rfs*12		T	
MN255	36	F	1	*AKT1 E17K*	ben-2		balanced	T	
MN330	54	F	1	*AKT1 E17K*				T	
MN353	77	F	1	*AKT1 E17K*				T	
MN368	50	M	1	*AKT1 E17K*				T	
MN396	36	F	1	*AKT1 E17K*				T	
MN315	76	F	1	*KLF4 K409Q*	ben-2		balanced	T	
MN331	73	F	1	*KLF4 K409Q*				T	
MN348	51	F	1	*KLF4 K409Q*				T	
MN377	63	F	1	*KLF4 K409Q*				T	
MN380	81	F	1	*KLF4 K409Q*				T	
MN428	42	F	2	NF2	int-A	NM_181830:exon10:c.1006delA:p.T336Rfs*7	chr 1, 6, 8, 10, 17p, 19, 22q loss		T, 2D, 3D
MN251	62	F	2	NF2	ben-1	NM_000268:exon1:c.108dupT:p.C37Lfs*12	chr 22q loss only		T, 2D, 3D
MN490	72	M	1	NF2	ben-1	NM_181830:exon2:c.136_157del:p.E46Rfs*38	chr 22q loss only		T, 2D, 3D
MN486	55	M	1	*AKT1 E17K*					T, 2D, 3D
MN493	54	F	1	NF2	ben-1	NM_016418:exon7:c.600-1G>C	chr 22q loss only		T
MN566	62	F	1	NF2	ben-1	NM_181830:exon11:c.1170delG:p.E391Rfs*11	chr 22q loss only	T, 3D	T
MN525	75	M	1	NF2	ben-1	NM_181830:exon7:c.600dupC:p.K201Qfs*3	chr 1q, 19, 22q loss	T, 3D	T, 2D, 3D
MN567	61	F	1	NF2		NM_181830:exon2:c.197_198del:p.K66Ifs*3		T, 3D	
MN573	70	F	1		ben-1		chr 19, 22q loss	T, 3D	
MN578	59	F	1	NF2	ben-1	NM_181830:exon5:c.C385T:p.Q129X	chr 22q loss only	T, 3D	T, 2D, 3D
MN580	54	F	2		int-A		chr 1p, 18q loss, chr 5, 20 gain	T, 3D	T
MN582	64	F	2	*AKT1 E17K*	ben-3		balanced	T, 3D	T, 2D, 3D
MN595	56	F	1	NF2	ben-1	NM_016418:exon8:c.810+1G>A	chr 22q loss only	T, 3D	T, 2D, 3D
MN598	81	F	1	*KLF4 K409Q*	ben-2		balanced		T, 2D, 3D
MN602	58	M	1		ben-1		chr 14q, 18, 22q loss	T, 3D	T, 2D, 3D
MN603	67	M	2	NF2		NM_000268:exon2:c.130delA:p.K44Rfs*79	chr 1p, 7q, 14q, 19q, 22q loss, chr 1q gain	T, 3D	
MN605	64	F	2		ben-1		chr 22q loss only	T, 3D	T, 2D, 3D
MN609	62	F	1	NF2	int-A	NM_181830:exon6:c.A433T:p.K145X	chr 1p, 6q, 10q, 22q loss, chr 6p gain	T, 3D	T, 2D, 3D

MN610	30	F	1	*KLF4 K409Q*	ben-3		balanced	T, 3D	
MN611	78	F	1	NF2	ben-3	NM_016418:exon2:c.115-2A>C	chr 22q loss, chr 3 gain	T, 3D	T, 2D, 3D
MN613	64	F	1	NF2		NM_016418:exon7:c.600-1G>C	chr 1p, 18, 2q loss	T, 3D	T, 2D, 3D
MN630	58	M	2	NF2	int-A	NM_181830:exon10:c.1025_1034del:p.L342Rfs*11	chr 1p, 6, 14q, 22q loss	T	T
MN656	78	F	1	NF2		NM_181830:exon10:c.1083_1084del:p.R363Efs*48	chr 22q loss only	T, 3D	T, 2D, 3D
MN655	65	F	1	*KLF4 K409Q*	ben-2		balanced	T, 3D	T, 2D, 3D
MN658	58	F	1	*KLF4 K409Q*	ben-3		chr 2 loss only	T, 3D	T, 2D, 3D
MN663	54	F	2	*AKT1 E17K*	ben-2		balanced	T, 3D	T, 2D, 3D
MN669	63	F	1		ben-3		chr 5, 6, 10, 12, 17, 18, 20 gain		T
MN689	61	M	1	*AKT1 E17K*				T, 3D	
MN711	45	F	1	*KLF4 K409Q*				T, 3D	T, 2D, 3D
MN682	52	M	1	*AKT1 E17K*	ben-2		balanced	T, 3D	
MN702	64	M	1	*AKT1 E17K*	ben-2		balanced	T, 3D	T
MN840	29	M	2		mal		chr 1p, 3, 5q, 7p, 10p, 22q loss, chr 5p gain	T	
MN841	68	F	3		int-A		chr 1p, 4, 10q, 14q, 18, 22q loss	T	
MN842	79	F	3		int-A		chr 1p, 10, 14q, 18, 22q loss	T	
MN843	61	F	3		mal		chr 1p, 2p, 3p, 4, 7p, 8p, 9p, 10p, 14q, 18, 22q loss, chr 1q, 7q, 9q, 20 gain	T	
MN844	57	M	2		mal		chr 1p, 4p, 5q, 6q, 8p, 22q loss, chr 10p, 11q gain	T	
MN845	76	M	1		mal		chr 1p, 3p, 6q, 7p, 19p, 22q loss	T	
MN846	46	M	1		mal		chr 1p, 2p, 6q, 11p, 17p, 22q loss	T	
MN847	48	M	3		mal		chr 1p, 7p, 10p, 18, 22q loss, chr 5, 7q, 12q, 13q, 14q, 17q, 20 gain	T	
MN848	55	M	2		mal		chr 1p, 2, 6q, 12p, 16, 22q loss, chr 17q gain	T	
MN849	58	M	2		mal		chr 1p, 4, 6q, 7p, 10q, 14q, 17p, 22q loss	T	

M: male; F: female; MC: methylation class; CNVs: copy number variations; mIHC: multiplex immunohistochemistry; RNA-seq: RNA sequencing; T: tissue; 2D: monolayer cells; 3D: spheroids

Supplementary Table 2. Oligonucleotide details

a. KASP™ genotyping primers

**Mutation**	**Primer Allele FAM**	**Primer Allele HEX**	**Primer Common**	**Allele FAM**	**Allele HEX**	**WT genotype**
AKT1 E17K (SNP: rs121434592)	CACCCGCACGTC TGTAGGGA	ACCCGCACGTCT GTAGGGG	GTGGCCGCCAGG TCTTGATGTA	A	G	GG
KLF4 K409Q	GTGCCTTGATGG GAACTCTT	GTGCCTTGAGAT GGGAACTCTG	GATTACGCGGGC TGCGGCAAAA	A	C	AA

b. KASP™ genotyping PCR condition

**Initial denaturation**	**Touchdown**	**Amplification**	**Final elongation**	**Amplification**	**Final elongation**
95°C for 15 min	10 cycles:<br>94°C for 15 s<br>61°C for 1 min	26 cycles:<br>94°C for 20 s<br>55°C for 1 min	37°C for 1 min	5 cycles:<br>94°C for 20 s<br>57°C for 1 min	37°C for 1 min

c. qPCR primers

**Name**	**TaqMan Assay ID**
CD68	Hs00154355_m1
IL6	Hs00174131_m1
IL10	Hs00961622_m1
TNF	Hs00174128_m1
CSF1	Hs00174164_m1
TGFB1	Hs00998133_m1
GAPDH	Hs02786624_g1

d. qPCR condition

**Initial denaturation**	**Amplification**
95°C for 2 min	45 cycles:<br>95°C for 15 s<br>60°C for 1 min

Supplementary Table 3. Antibodies details

a. Primary antibodies and corresponding Opal fluorescence for mIHC

Primary antibody	Company	Cat. No.	Dilution	Opal fluorescence	Staining order	RRID
CD68	Agilent	M0876	1:100	Opal 690	5	AB_2074844
CD163	Abcam	ab265592	1:100	Opal 480	4	AB_3674755
TMEM119	Atlas Antibodies	AMAb91528	1:500	Opal 520	3	AB_2797214
P2RY12	Merck	HPA014518	1:100	Opal 620	1	AB_2669027
CD3	Abcam	ab16669	1:150	Opal 570	2	AB_443425

b. Primary and secondary antibodies for WB

Primary antibody	Species	Company	Cat. No.	Dilution	RRID
CD68	Rabbit	Abcam	ab213363	1:1000	AB_2801637
CD163	Rabbit	Abcam	ab182422	1:500	AB_2753196
TMEM119	Mouse	Atlas Antibodies	AMAb19528	1:500	AB_2797214
Merlin	Rabbit	Cell Signaling Technology	6995	1:1000	AB_10828709
Phospho-Merlin	Rabbit	Cell Signaling Technology	13281	1:1000	AB_2650552
GAPDH	Mouse	Abcam	ab8245	1:5000	AB_2107448

Secondary antibody	Type	Company	Cat. No.	Dilution	RRID
Goat anti-rabbit	HRP-conjugated	Bio-Rad	1706515	1:10000	AB_11125142
Goat anti-mouse	HRP-conjugated	Bio-Rad	1706516	1:10000	AB_2921252

c. Primary and secondary antibodies for ICC

Primary antibody	Species	Company	Cat. No.	Dilution	RRID
Alexa Fluor™ 488-conjugated CD68	Mouse	Abcam	ab222914	1:50	AB_3674756
CD68	Rabbit	Abcam	ab213363	1:100	AB_2801637
CD163	Rabbit	Abcam	ab182422	1:100	AB_2753196
TMEM119	Mouse	Atlas Antibodies	AMAb91528	1:50	AB_2797214
Alexa Fluor™ 488-conjugated IgG1	Mouse	Abcam	ab234075	1:50	AB_3674757
CD68	Mouse	Abcam	ab955	1:50	AB_307338
Ki67	Mouse	Agilent	M7240	1:100	AB_2142367

Secondary antibody	Type	Company	Cat. No.	Dilution	RRID
Goat anti-rabbit	Alexa Fluor™ 568-conjugated	Thermo Fisher Scientific	A11011	1:500	AB_143157
Goat anti-mouse	Alexa Fluor™ 488-conjugated	Thermo Fisher Scientific	A11001	1:500	AB_2534069
