# Supplementary material for: Tumour-associated macrophage infiltration differs in meningioma genotypes, and is important in tumour dynamics
Source: J Exp Clin Cancer Res. 2025 May 27;44:162. doi: 10.1186/s13046-025-03419-2 (PMC12107748; doi:10.1186/s13046-025-03419-2)
Supplement: Supplementary file 2 — Supplementary Material 2: Additional file 2 [file 13046_2025_3419_MOESM2_ESM.pdf]

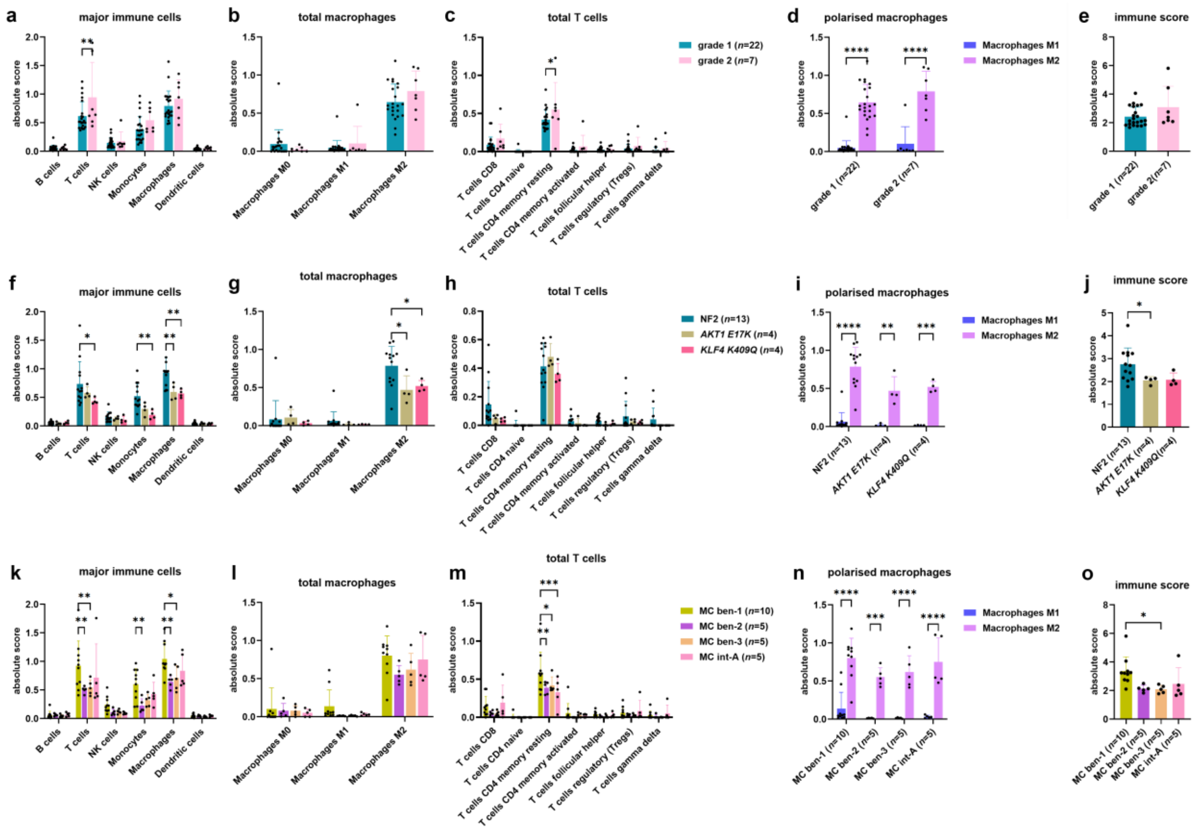

Supplementary Fig. 1 Immune profile of meningioma tissue with different classifications analysed by CIBERSORTx using bulk RNA-seq data. (a-c) Analyses of different WHO grades showing the proportion of **(a)** major immune cells, **(b)** total macrophages, **(c)** total T cells. **(d)** Comparison between polarised M1 and M2 macrophages across grades. **(e)** Comparison of immune score between grade 1 and 2. **(f-h)** Analyses of different genotypes showing the proportion of **(f)** major immune cells, **(g)** total macrophages, **(h)** total T cells. **(i)** Comparison between polarised M1 and M2 macrophages across genotypes. **(j)** Comparison of immune score among genotypes. **(k-o)** Analyses of different MCs showing the proportion of **(k)** major immune cells, **(l)** total macrophages, **(m)** total T cells. **(n)** Comparison between polarised M1 and M2 macrophages across MCs. **(o)** Comparison of immune score among MCs. Total macrophages equal to TAMs, M1 macrophages equal to M1-like TAMs, and M2 macrophages equal to M2-like TAMs. There is no grade 3 or MC mal samples for bulk RNA-seq. Statistical analyses were performed using two-way ANOVA with Sidak's **(a-d, i, n)** or Tukey's **(f-h, k-m)** multiple comparisons test for cell type proportions, and unpaired Student's t-test **(e)**, one-way ANOVA with Dunnett's T3 multiple comparisons test **(j)** or nonparametric test with Dunn's multiple comparisons test **(o)** for immune score as appropriate. *p<0.05, **p<0.01, ***p<0.001, ****p<0.0001.

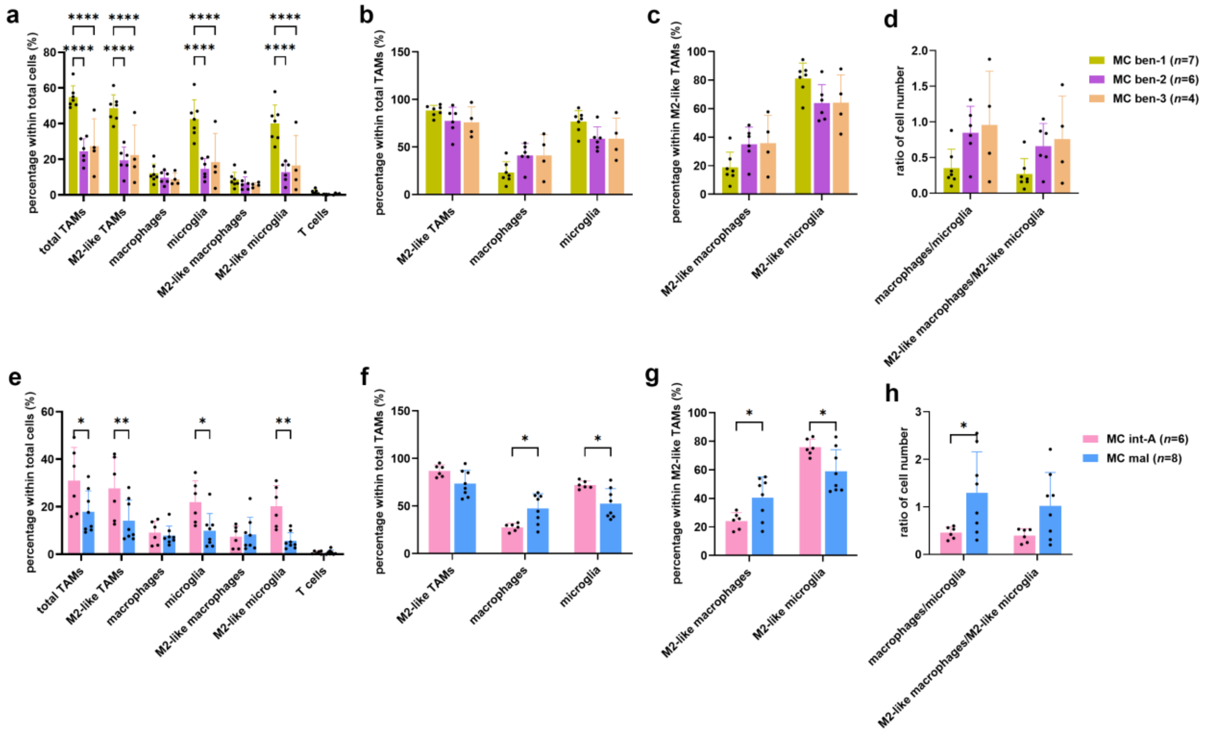

Supplementary Fig. 2 TME landscape of meningioma tissue with benign MCs and malignant MCs analysed by mIHC data. (a) Proportion of various immune cells in the total cell population in benign MCs. **(b)** Proportion of various subtypes of TAMs within total TAMs in benign MCs. **(c)** Proportion of M2-like subtypes within M2-like TAMs in benign MCs. **(d)** Ratio of cell number of macrophages to microglia and M2-like macrophages to M2-like microglia in benign MCs. **(e)** Proportion of various immune cells in the total cell population in malignant MCs. **(f)** Proportion of various subtypes of TAMs within total TAMs in malignant MCs. **(g)** Proportion of M2-like subtypes within M2-like TAMs in malignant MCs. **(h)** Ratio of cell number of macrophages to microglia and M2-like macrophages to M2-like microglia in malignant MCs. Statistical analyses were performed using two-way ANOVA with Tukey's **(a-d)** or Sidak's **(e-h)** multiple comparisons test. *p<0.05, **p<0.01, ***p<0.001, ****p<0.0001.

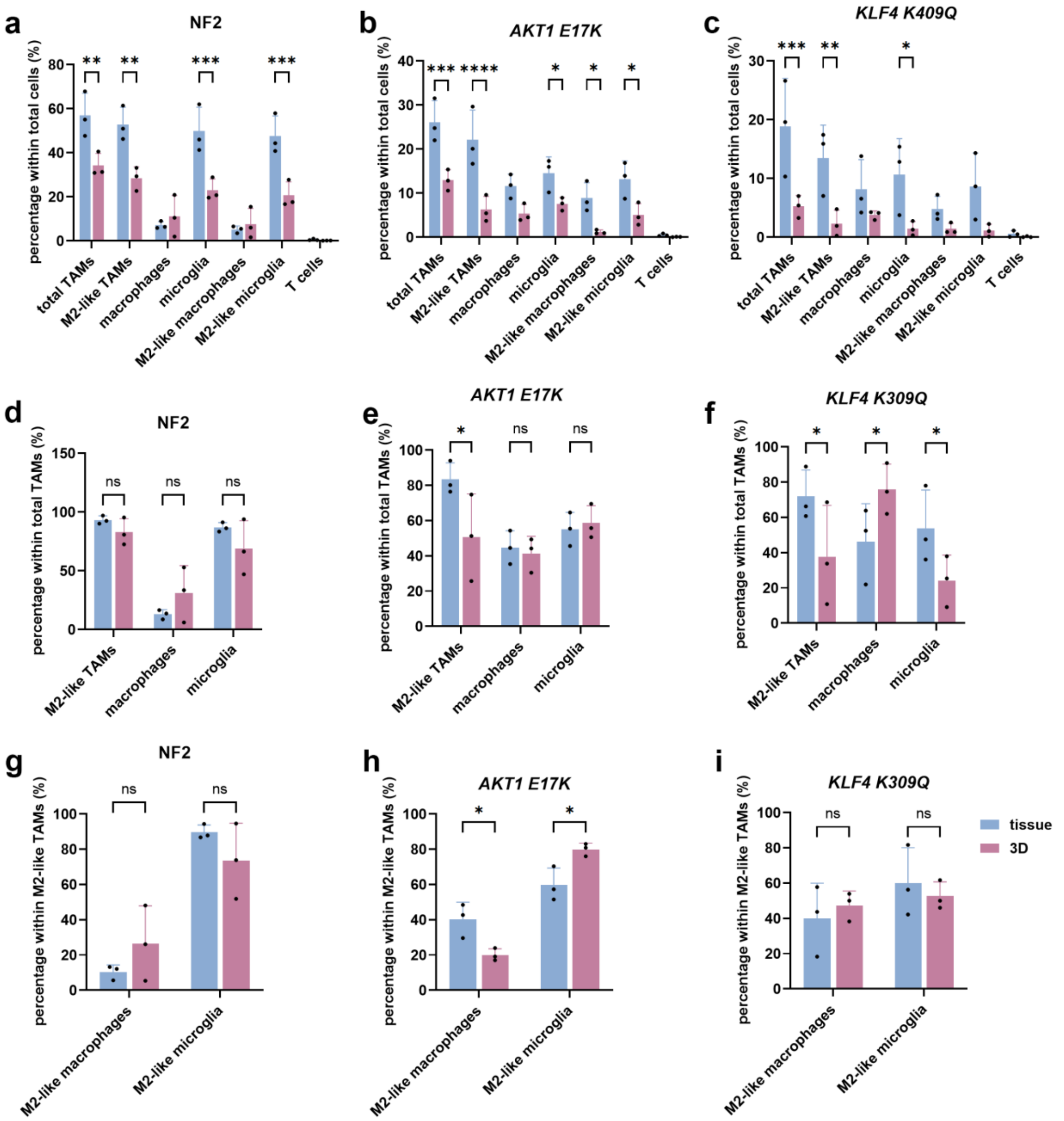

Supplementary Fig. 3 Comparisons of TME landscape in meningioma tissue and matched 3D samples with different genotypes analysed by mIHC data. (a-c) Proportion of various immune cells in the total cell population in different genotypes compared parental tissue and matched 3D. **(d-f)** Proportion of various subtypes of TAMs within total TAMs in different genotypes compared parental tissue and matched 3D. **(g-i)** Proportion of various M2-like subtypes within M2-like TAMs in different genotypes compared parental tissue and matched 3D. Statistical difference was analysed using mixed-effects analysis with Sidak's multiple comparisons test. *p<0.05, **p<0.01, ***p<0.001, ****p<0.0001.

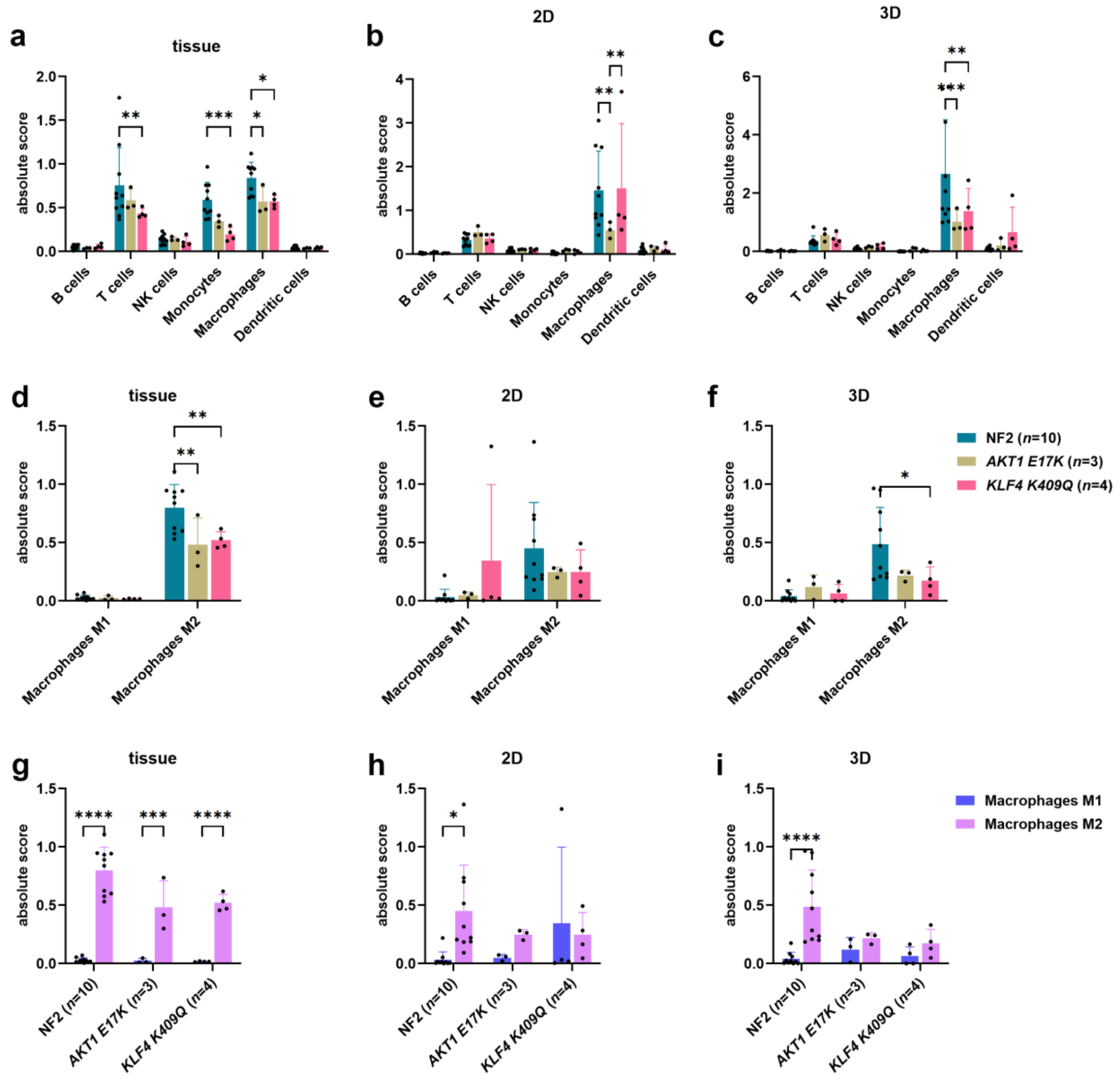

Supplementary Fig. 4 Comparisons of immune cells in meningioma tissue, matched 2D and 3D samples with different genotypes analysed by CIBERSORTx using bulk RNA-seq data. (a-c) Proportion of major immune cells in matched tissue, 2D and 3D samples compared among different genotypes. **(d-f)** Proportion of polarised M1 and M2 macrophages in matched tissue, 2D and 3D samples compared among different genotypes. **(g-i)** Comparison of the proportion of polarised M1 and M2 macrophages in matched tissue, 2D and 3D samples among different genotypes. Statistical analyses were performed using two-way ANOVA with Tukey's **(a-f)** or Sidak's **(g-i)** multiple comparisons test. *$p<0.05$, **$p<0.01$, ***$p<0.001$, ****$p<0.0001$.

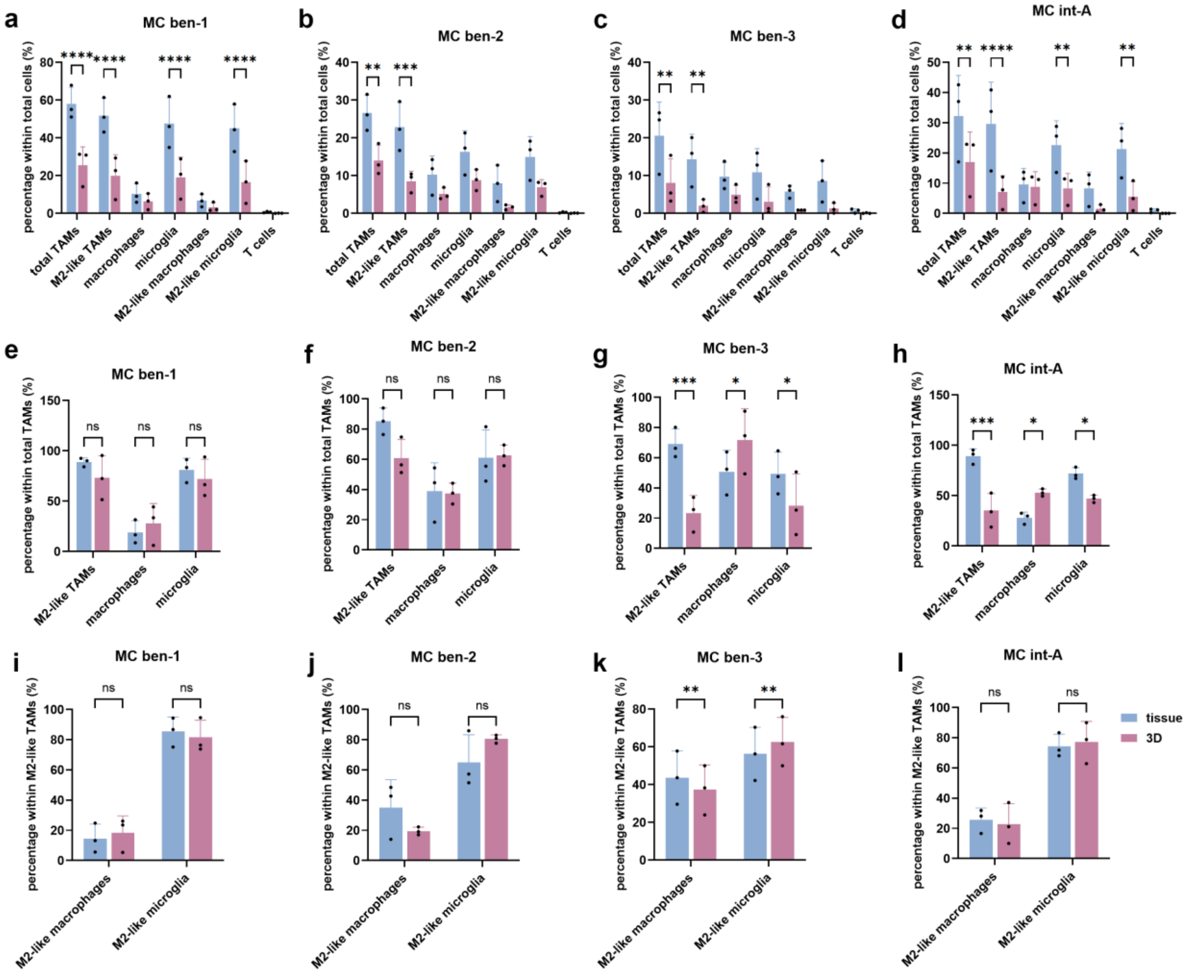

Supplementary Fig. 5 Comparisons of TME landscape in meningioma tissue and matched 3D samples with different MCs analysed by mIHC data. (a-d) Proportion of various immune cells in the total cell population in different MCs compared parental tissue and matched 3D. **(e-h)** Proportion of various subtypes of TAMs within total TAMs in different MCs compared parental tissue and matched 3D. **(i-l)** Proportion of various M2-like subtypes within M2-like TAMs in different MCs compared parental tissue and matched 3D. Statistical differences were analysed using mixed-effects analysis with Sidak's multiple comparisons test. *p<0.05, **p<0.01, ***p<0.001, ****p<0.0001.

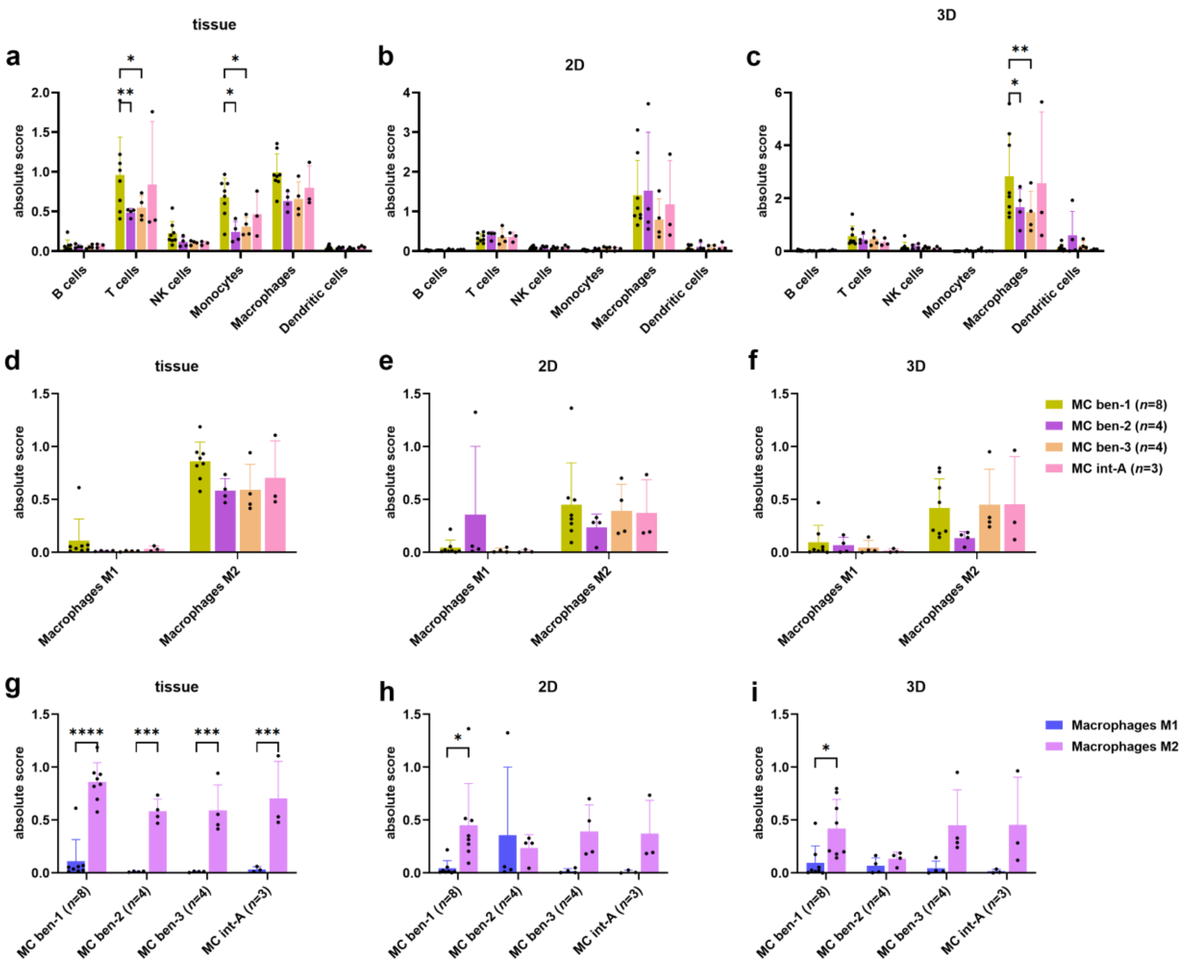

Supplementary Fig. 6 Comparisons of immune cells in meningioma tissue, matched 2D and 3D samples with different MCs analysed by CIBERSORTx using bulk RNA-seq data. **(a-c)** Proportion of major immune cells in matched tissue, 2D and 3D samples compared among different MCs. **(d-f)** Proportion of polarised M1 and M2 macrophages in matched tissue, 2D and 3D samples compared among different MCs. **(g-i)** Comparison of the proportion of polarised M1 and M2 macrophages in matched tissue, 2D and 3D samples across different MCs. Statistical differences were performed using two-way ANOVA with Tukey's **(a-f)** or Sidak's **(g-i)** multiple comparisons test. **p*<0.05, ***p*<0.01, ****p*<0.001, *****p*<0.0001.

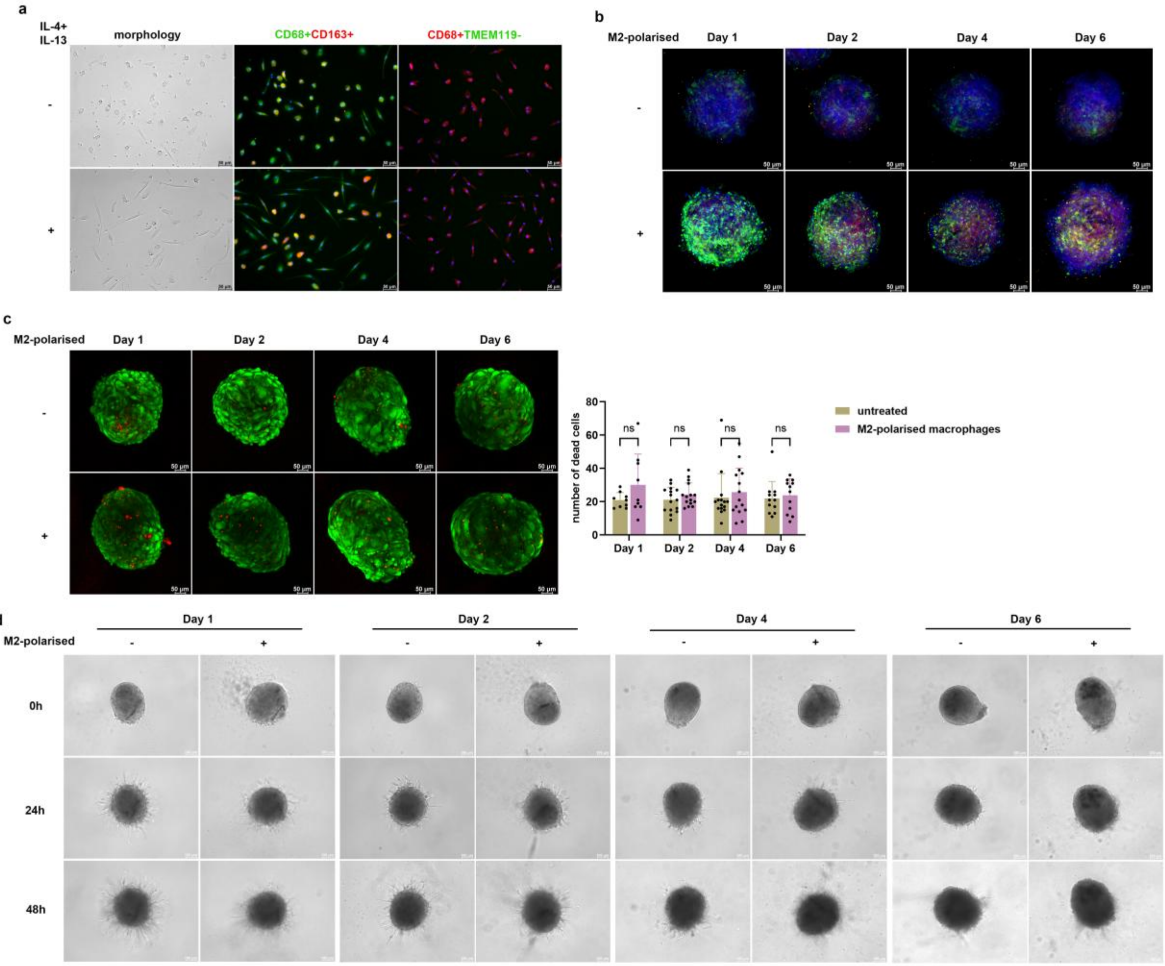

Supplementary Fig. 7 The 3D co-culture model to show the function of M2-polarised macrophages in TME. (a) Validation of M2-polarised macrophages was performed by ICC and cell morphology. M2-polarised macrophages were differentiated from PBMCs isolated from the patient's blood using M-CSF and polarised with IL-4 and IL-13. The macrophages were characterised by co-expression of CD68 (green) and CD163 (red), and CD68 expression (red) but negative for TMEM119. Images were taken at 200x magnification using a Leica IM8 microscope. **(b)** Validation of M2-polarised macrophages infiltration in spheroids was assessed by ICC, showing co-expression of CD68 (green) and CD163 (red), with nuclei stained by DAPI (blue) compared between untreated and treated M2-polarised macrophages in 3D models. Images were captured over time using Leica Stellaris at 200× magnification. **(c)** Tumour cell viability was assessed by a live/dead cell assay, staining the 3D model with Calcein AM (live cells, green) and Ethidium homodimer-1 (dead cells, red) over time. Images were captured using Leica Stellaris at 200× magnification. The number of dead cells was counted to compare between untreated and treated M2-polarised macrophages in 3D models over time. Statistical analyses were performed using two-way ANOVA with Sidak's multiple comparisons test. **(d)** Tumour cell invasion was performed using Matrigel assay. Matrigel was applied on top of the 3D model at different time points from 0 h to 48 h. Images were captured every 24 h using Leica IM8 at 100× magnification.

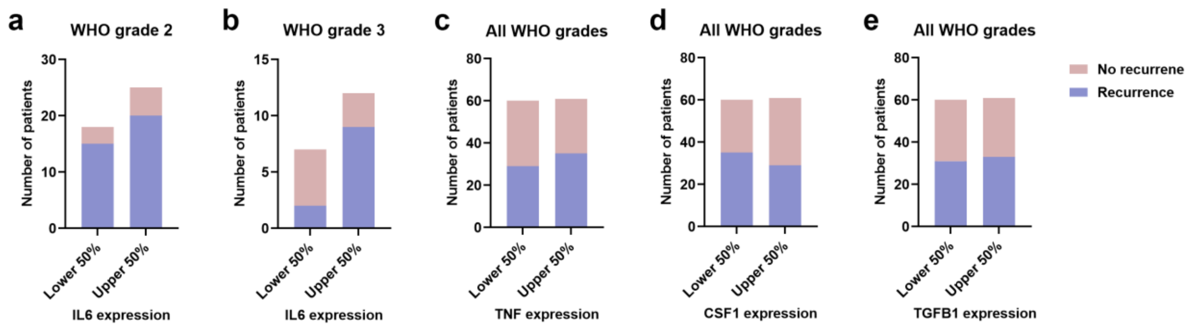

Supplementary Fig. 8 Correlations between macrophage-related cytokines and tumour recurrence in cBioportal analysis. (a) *IL6* expression in WHO grade 2 patients (p>0.9999), **(b)** *IL6* expression in WHO grade 3 patients (p=0.0739), **(c)** *TNF* expression in all patients (p=0.3648), **(d)** *CSF1* expression in all patients (p=0.2762), **(e)** *TGFB1* expression in all patients (p=0.8562). Statistical analyses were performed using Fisher's exact test.
